# Transcriptome-based analysis of the effects of salicylic acid and high light on lipid and astaxanthin accumulation in *Haematococcus pluvialis*

**DOI:** 10.1186/s13068-021-01933-x

**Published:** 2021-04-01

**Authors:** Qunju Hu, Danqiong Huang, Anguo Li, Zhangli Hu, Zhengquan Gao, Yongli Yang, Chaogang Wang

**Affiliations:** 1 Shenzhen Key Laboratory of Marine Bioresource and Eco-Environmental Science, Shenzhen Engineering Laboratory for Marine Algal Biotechnology, Guangdong Provincial Key Laboratory for Plant Epigenetics, College of Life Sciences and Oceanography, Shenzhen University, Nanshan District, Xueyuan Road No. 1066, Shenzhen, 518060 Guangdong People’s Republic of China; 2 Key Laboratory of Optoelectronic Devices and Systems of Ministry of Education and Guangdong Province, College of Optoelectronic Engineering, Shenzhen University, Shenzhen, 518060 China; 3 College of Food Engineering and Biotechnology, Hanshan Normal University, Chaozhou, 521041 China; 4 College of Life Sciences, Shandong University of Technology, Zibo, 255049 China

**Keywords:** *Haematococcus pluvialis*, Salicylic acid, High light stress, Transcriptome sequencing, Astaxanthin biosynthesis, Fatty acid biosynthesis

## Abstract

**Background:**

The unicellular alga *Haematococcus pluvialis* has achieved considerable interests for its capacity to accumulate large amounts of triacylglycerol and astaxanthin under various environmental stresses. To our knowledge, studies focusing on transcriptome research of *H. pluvialis* under exogenous hormones together with physical stresses are rare. In the present study, the change patterns at transcriptome level were analyzed to distinguish the multiple defensive systems of astaxanthin and fatty acid metabolism against exogenous salicylic acid and high light (SAHL) stresses.

**Results:**

Based on RNA-seq data, a total of 112,463 unigenes and 61,191 genes were annotated in six databases, including NR, KEGG, Swiss-Prot, PFAM, COG and GO. Analysis of differentially expressed genes (DEGs) in KEGG identified many transcripts that associated with the biosynthesis of primary and secondary metabolites, photosynthesis, and immune system responses. Furthermore, 705 unigenes predicted as putative transcription factors (TFs) were identified, and the most abundant TFs families were likely to be associated with the biosynthesis of astaxanthin and fatty acid in *H. pluvialis* upon exposure to SAHL stresses. Additionally, majority of the fifteen key genes involved in astaxanthin and fatty acid biosynthesis pathways presented the same expression pattern, resulting in increased accumulation of astaxanthin and fatty acids in single celled *H. pluvialis*, in which astaxanthin content increased from 0.56 ± 0.05 mg·L^−1^ at stage Control to 0.89 ± 0.12 mg·L^−1^ at stage SAHL_48. And positive correlations were observed among these studied genes by Pearson Correlation (PC) analysis, indicating the coordination between astaxanthin and fatty acid biosynthesis. In addition, protein–protein interaction (PPI) network analysis also demonstrated that this coordination might be at transcriptional level.

**Conclusion:**

The results in this study provided valuable information to illustrate the molecular mechanisms of coordinate relations between astaxanthin and fatty acid biosynthesis. And salicylic acid might play a role in self-protection processes of cells, helping adaption of *H. pluvialis* to high light stress.

**Supplementary Information:**

The online version contains supplementary material available at 10.1186/s13068-021-01933-x.

## Highlights

- Increased accumulation of carotenoids, astaxanthin and fatty acids in individual cells were observed according to SAHL treatment.
- High quality RNA-seq analysis identified 61,191 genes upon exposure to SAHL stresses.
- Positive correlations between fifteen genes involved in carotenoid and fatty acid biosynthesis were observed, and PPI network analysis indicated that ACP gene might play a role in coordination between these two modules.
- Salicylic acid might play a role in self-protection processes, helping adaption of *H. pluvialis* cells to high light stress.

## Background

*Haematococcus pluvialis* is a unicellular microalga that has been widely considered as a rich, natural resource of the high-value ketocarotenoid astaxanthin, and astaxanthin can be dramatically accumulated in the cells upon exposure to various stresses, such as low levels of oxygen, low or high levels of chemical substances, high temperature and high light intensity [1–5]. High concentration of astaxanthin in *H. pluvialis* is considered to be part of the self-protection process of cells in stressful environments [6]. Salicylic acid (SA) is produced by a wide range of prokaryotic and eukaryotic organisms as a secondary metabolite, and acts as an immune response signaling molecule in plants, which has been shown to be beneficial for them in both optimal and stress environments [7–10]. SA was suggested to constitute molecular signals in the astaxanthin biosynthesis pathway, and it could be used as a potential regulator for astaxanthin production in *H. pluvialis* [4, 11–16]. Furthermore, higher transcriptional expression levels of the carotenogenesis genes (e.g., IPI1, IPI2, PSY, PDS, LYC, BKT2, CRTR-B and CRTO) could be induced by SA, and higher astaxanthin productivity in *H. pluvialis* was also reported as a result of SA inductions [4, 15]. However, the study on the regulatory and physiological roles in which SA plays in *H. pluvialis* responsing to environmental stress factors (e.g., high light intensity) at the level of transcription is limited and relevant pathways are also not fully documented.

Transcriptome sequencing is considered to be an efficient and essential approach to obtain functional genomics information of microalgae [17]. In fact, transcriptome research of microalgae has made progress in recent years [5, 15–21]. For example, transcriptome analysis has been used as a powerful tool to identify important genes and key pathways for the biosynthesis of important metabolites, including, but not limited to, astaxanthin, fatty acids and triacylglycerol in microalgae [5, 17, 18, 21–23]. The gene transcript and metabolic changes of *H. pluvialis* upon exposure to various stresses (including light intensity, temperature, salinity, hormone, iron and inorganic carbon) have also been discussed in previous studies [3–5, 15, 24–27]. Nevertheless, little is known about the effects of hormones on the response of *H. pluvialis* to other environmental stresses, especially the genetic details and regulation mechanisms resulting in accumulation of astaxanthin and fatty acids. To our knowledge, works focusing on transcriptome analyses of *H. pluvialis* in response to exogenous hormones together with physical stresses is rare. Therefore, a genome-wide investigation, accompanied by differential gene expression analysis, would provide important information in understanding and genetic engineering of *H. pluvialis* for astaxanthin and fatty acids production.

For the inaccessible of genome sequence to quantify the transcriptome, global transcriptomic analysis of the astaxanthin production microalga *H. pluvialis* was slowly investigated. An exploration for molecular mechanism of *H. pluvialis* in regulation of astaxanthin and fatty acid biosyntheses upon exposure to SA and high light (HL) stresses is evaluated by transcriptome analysis in this study. Based on our previously reported genome database of *H. pluvialis* [28], a detailed analysis of the genetic information for the stress response mechanism was determined. Clean unigenes of the microalga *H. pluvialis* were assembled using the reported genome data and then annotated based on the information from various databases, including NR, KEGG, Swiss-Prot, PFAM, KOG and GO. The transcriptome sequences will provide a valuable genomic resource of information to further understand the molecular mechanism of hormone (SA) on regulating the response of *H. pluvialis* to high light stress, laying a foundation for insight into the mechanism of astaxanthin accumulation in this alga, and may also provide guidance on industrial production of astaxanthin.

## Results and discussion

### Morphological changes of alga cells in responding to SAHL stresses

**Fig. 1:**
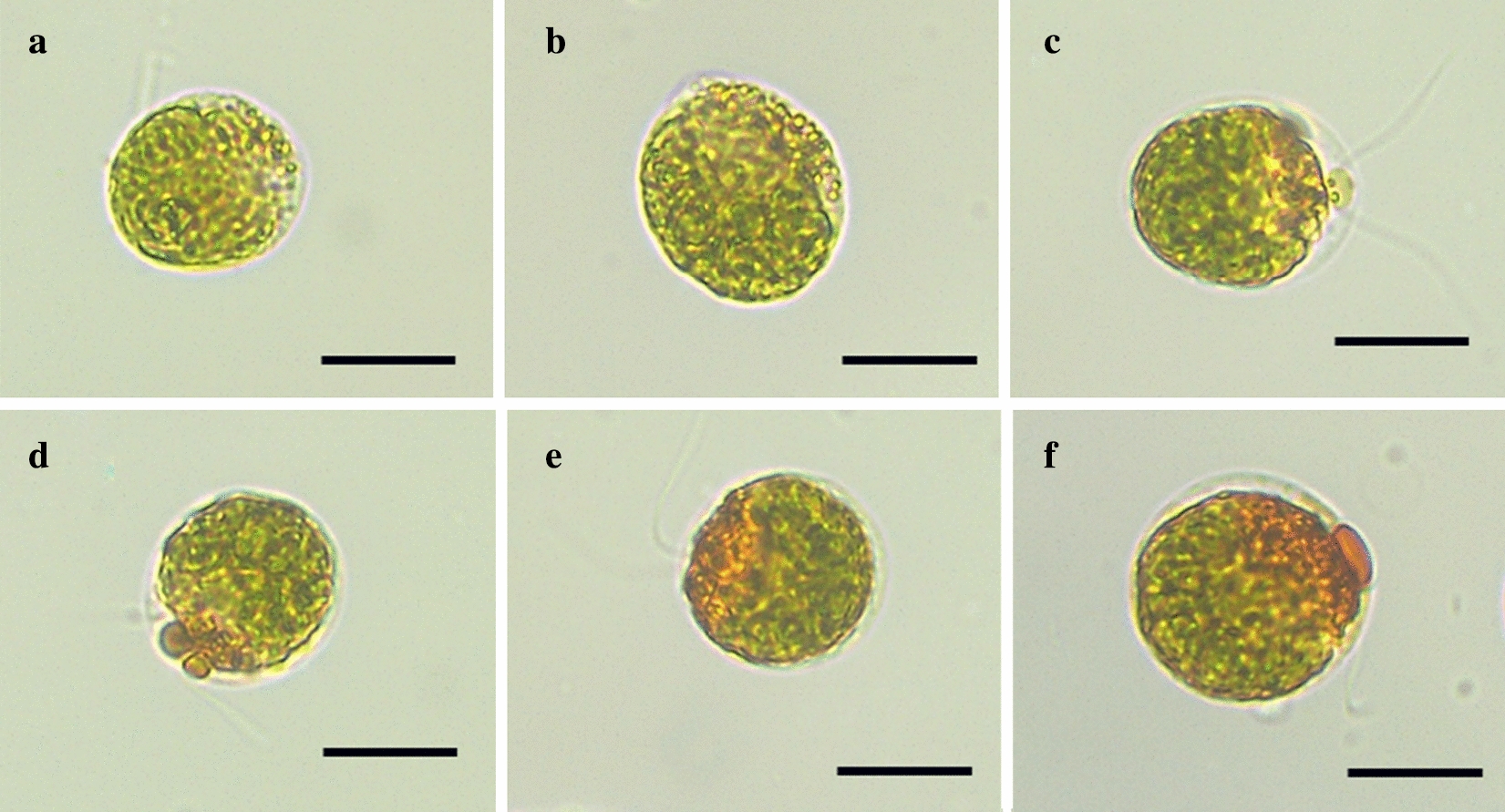
Microscopic images of *H. pluvialis* cells at different SAHL treatment stages. **a**–**f**) represent the samples treated with SAHL for 0 h (Control), 1 h (SAHL_1), 6 h (SAHL_6), 12 h (SAHL_12), 24 h (SAHL_24) and 48 h (SAHL_48), respectively. Scale bar = 20 μm

Morphology changes of *H. pluvialis* at different treatment stages were observed in this study (Fig. 1). As shown in Fig. 1, astaxanthin, which represented in orange red color, was visually increased inside the cells along with the time course of SAHL treatment (Control, SAHL_1, SAHL_6, SAHL_12, SAHL_24 and SAHL_48) through an optical microscope. Microscopy observations revealed that the color of the cells was changed from green to lim-green from stage Control to stage SAHL_48, and the orange red partial of the cells were perceived obviously increased. Interestingly, flagella lost were not observed in this study.

The pattern of the batch growth is measured in Table 1. In this study, all treatments started with the same cell density (CD) of 9.92 ± 0.91 × 10^4^ cell·mL^−1^. However, CD under SAHL treatments maintain unchanged during the first 12 h and then gradually decreased along with the time course due to increasing dead cells and cell debris in the cultures. Therefore, dry cell weights (DCW) of all these treatments remain unchanged along with the time course, as revealed in Table 1, which maintained around 0.13 g·L^−1^. The carotenoid contents (CC) and single cell carotenoid contents (SCCC) of the treatment stages displayed a similar change tendency, which showed the lowest level at stage SAHL_6, and increased along with time course of SAHL treatment. CC and SCCC at stage SAHL_48 were 117.69 ± 7.14% and 224.67 ± 15.39% higher than those during Control stage. As shown in Table 1, astaxanthin contents (AC) were significantly increased along with the extension of treatment time, and AC at stage SAHL_48 were 59.60 ± 15.61% higher than that during stage Control. Total fatty acids (TFA) with a basal level of 139.03 ± 23.23 mg·g^−1^ by dry cell weight was measured in this study, and remained more or less constant in the 2 day cultivation. No significant differences in fatty acids content to dry cell weight were observed. In this study, increased accumulations of carotenoids, astaxanthin and fatty acids in individual cells were observed similar to color change of the cells.

**Table 1:** Effects of SAHL treatments on growth measurements of *H. pluvialis*

Stages	Control	SAHL_1	SAHL_6	SAHL_12	SAHL_24	SAHL_48
Measurements						
CD	10.02 ± 0.12^a^	10 ± 0.84^a^	9.67 ± 1.23^a^	8.08 ± 0.53^b^	7.94 ± 1.05^b^	6.76 ± 0.29^c^
DCW	0.13 ± 0.01	0.13 ± 0.02	0.13 ± 0.02	0.12 ± 0.01	0.13 ± 0.02	0.15 ± 0.01
CC	0.49 ± 0.01^c^	0.46 ± 0.03^c^	0.40 ± 0.04^d^	0.56 ± 0.02^b^	0.58 ± 0.03^b^	1.07 ± 0.02^a^
SCCC	4.88 ± 0.04^c^	4.62 ± 0.40^c^	4.22 ± 0.61^d^	6.93 ± 0.53^b^	7.52 ± 1.38^b^	15.86 ± 0.66^a^
AC	0.56 ± 0.05^c^	0.64 ± 0.06^bc^	0.66 ± 0.05^bc^	0.76 ± 0.06^ab^	0.88 ± 0.12^a^	0.89 ± 0.12^a^
TFAC	154.56 ± 16.56	134.65 ± 11.25	140.88 ± 28.67	119.55 ± 9.26	138.39 ± 26.62	146.16 ± 18.62

Data are given as means ± S.D., n = 3. *CD* Cell Density (× 10^4^ cell·mL^−1^), *DCW* Dry Cell Weight (mg·L^−1^), *CC* Carotenoids Content (mg·L^−1^), *SCCC* Single Cell Carotenoids Content (pg·cell^−1^), *AC* Astaxanthin Content (mg·L^−1^), *TFAC*: Total Fatty acids Content (mg·g^−1^); Different Peer Data shoulder superscript lowercase letters indicate significant differences (*p* < 0.05) among treatments, and any of the same lowercase letters or letter said the difference was not significant (*p* > 0.05)

### RNA Sequencing, assembly and annotation analysis

In this study, the transcriptomic changes and gene expression profiling associated with carotenoid and fatty acid biosynthesis under exogenous salicylic acid and high light stresses were studied. To explore molecular basis of the morphological and growth differences in alga cells described above, transcriptome profiles were generated. In total, 6 libraries corresponding to the six SAHL treatment stage samples were constructed and analyzed. As shown in Table 2, over 7.78 GB clean data from each of the algal samples with Q20 higher than 97.91% and low quality reads rates lower than 0.03% were obtained by the high throughput RNA sequencing. GC contents of the sequences were ranging from 59.41% to 59.73%. The reads of RNA-Seq were aligned with the reference map of the newly assembled *H. pluvialis* genome [28], and mapping rate of these algal samples were all higher than 93.41% (Table 2). As obtained from the results in Table 2, the transcripts were expressed at similarity levels. The raw sequence data generated from this study have been deposited in the NCBI with the SRA accession number of PRJNA675306 (http://www.ncbi.nlm.nih.gov/sra/PRJNA675306).

**Table 2:** RNA sequencing and mapping results

Algal Samples	Raw reads	Clean reads	Clean bases (nt)	Error rate (%)	Q20 (%)	GC Content (%)	Mapping rate (%)
Control	51,078,628	50,323,266	7,453,145,620	0.02	98.03	59.41	93.87
SAHL_1	53,111,838	52,448,902	7,809,908,445	0.02	98.1	59.73	93.81
SAHL_6	48,123,996	47,487,934	7,078,178,206	0.03	97.91	59.54	93.41
SAHL_12	46,615,054	46,032,120	6,828,091,702	0.02	98.29	59.44	93.60
SAHL_24	49,554,888	48,744,408	7,204,906,648	0.03	97.92	59.72	93.57
SAHL_48	49,722,568	48,911,296	7,251,211,966	0.02	98.19	59.64	93.84

**Fig. 2:**
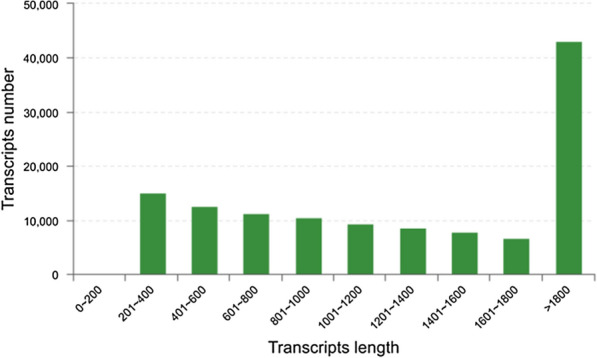
Results of de novo assembly about length distribution of transcripts

A total of 112,463 unigenes was generated by assembly of the reads. The length of the unigenes varied from 69 bp to 38,640 bp with an average of 1,410 bp and N50 of 2,102 bp, and a total length of 86,278,165 bp (Fig. 2). In general, 93,942; 93,711; 93,276; 93,536; 94,890 and 94,904 transcripts were found to be expressed in stages of Control, SAHL_1, SAHL_6, SAHL_12, SAHL_24 and SAHL_48, respectively. The numbers of identified transcripts in each sample, expressed in FPKMs, were shown in Additional file 1: Fig. S1A. It was revealed that approximately 55% of the expressed genes were in the 0.5–5 FPKM range, and 30% of the expressed genes were in the FPKM range of 5–100. Principal component analysis revealed that six samples could be clearly assigned to separate groups (Additional file 1: Fig. S1B), suggesting that the overall transcriptome profiling was different at each SAHL treatment stage in *H. pluvialis*.

The 112,463 unigenes and 61,191 genes were then annotated by BLAST using six databases, including: NR (NCBI non-redundant protein sequences), KEGG (KEGG Ortholog), Swiss-Prot (A manually annotated and reviewed protein sequence database), Pfam (Protein family), COG (Clusters of Orthologous Groups of proteins), and GO (Gene Ontology). Specifically, 32,250 genes (52.70%), 16,796 genes (27.45%), 19,505 (31.88%), 24,188 genes (39.53%), 24,864 genes (40.63%) and 24,206 genes (39.56%) were annotated in NR, KEGG, Swiss-Prot, PFAM, COG and GO, respectively (Table 3).

**Table 3:** Summary of the function annotation results in six public protein databases for the *H. pluvialis* unigenes

Annotated databases	Number of genes	Percentage (%)
Annotated in NR	32,250	52.70
Annotated in KEGG	16,796	27.45
Annotated in Swiss-Prot	19,505	31.88
Annotated in PFAM	24,188	39.53
Annotated in COG	24,862	40.63
Annotated in GO	24,206	39.56

**Fig. 3:**
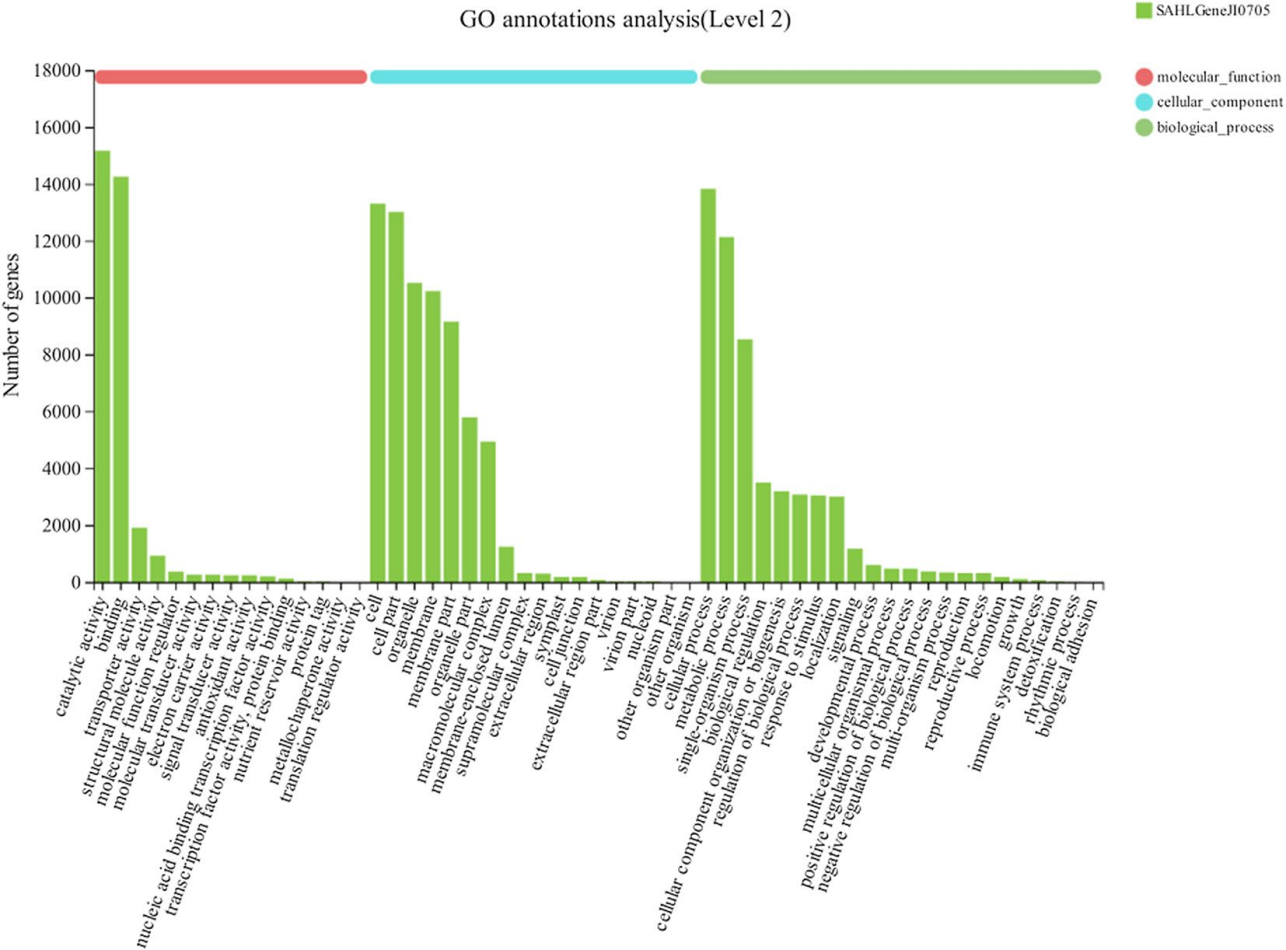
Gene function classification of all unigenes annotated by Gene Ontology (GO). GO classification were represented in histogram

Based on homologous genes, 24,206 unigenes were categorized into 55 GO terms in three non‐overlapping domains, including biological process, cellular component, and molecular function (Fig. 3). It was clearly displayed that the dominant distributions were from “catalytic activity”, “binding”, “cell”, “cell part”, “organelle”, “membrane”, “membrane part”, “cellular process”, “metabolic process”, and “single-organism process” terms.

**Fig. 4:**
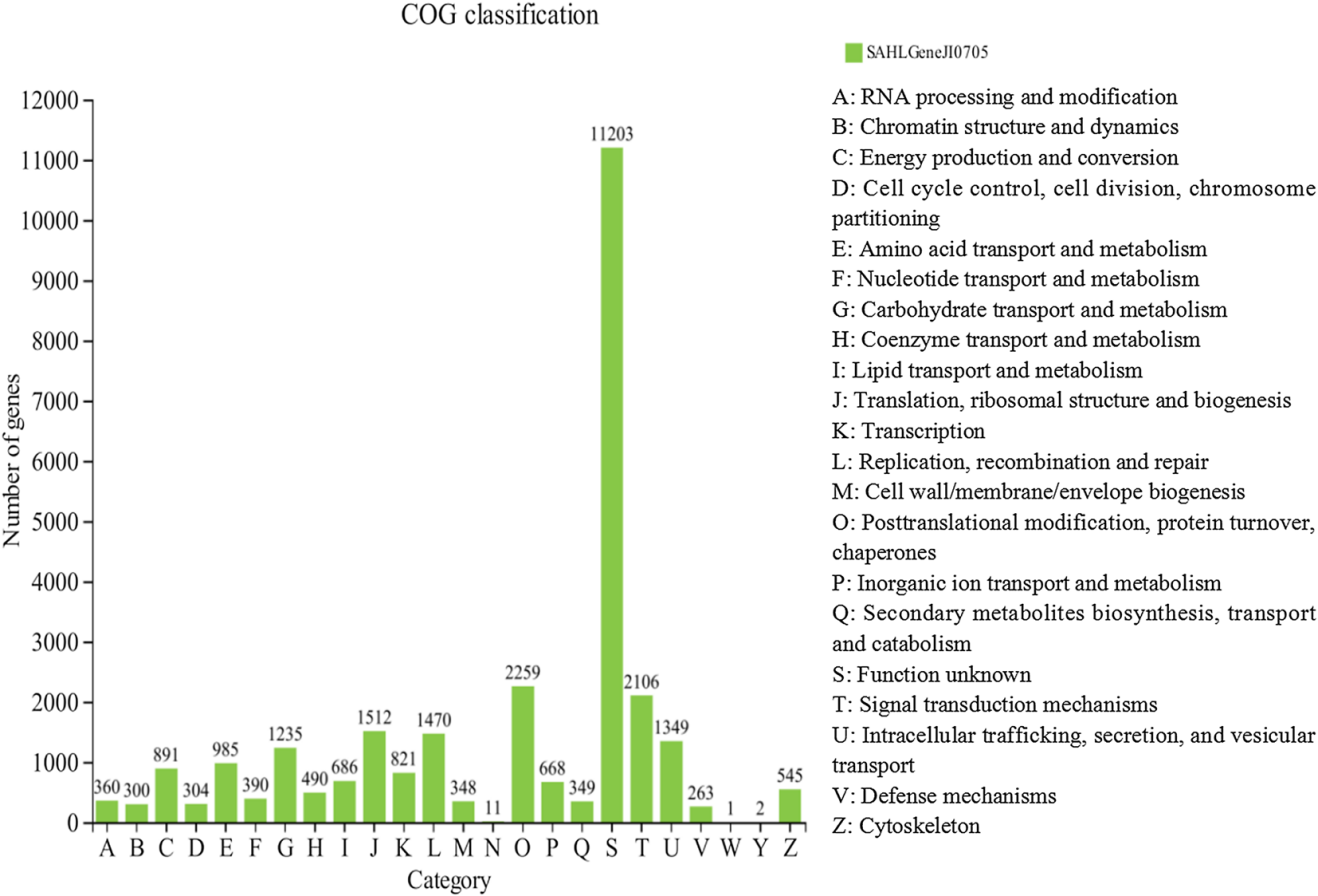
Orthologous groups (COG) functional classification of the unigenes. COG classification were represented in histogram

COG classification of the unigenes was performed to evaluate further the integrality of our transcriptomic libraries and the effectiveness of the annotation process, and the results were shown in Fig. 4. Generally, 24,862 unigenes were categorized into 24 COG categories, in which “Function unknown” was the highest occupied cluster (11,203, 39.24%) followed by “Posttranslational modification, protein turnover, chaperones” (2,259, 7.91%), “Signal transduction mechanisms” (2,106, 7.38%), “Translation, ribosomal structure and biogenesis” (1,512, 5.30%), and “Replication, recombination and repair” (1,470, 5.15%).

**Fig. 5:**
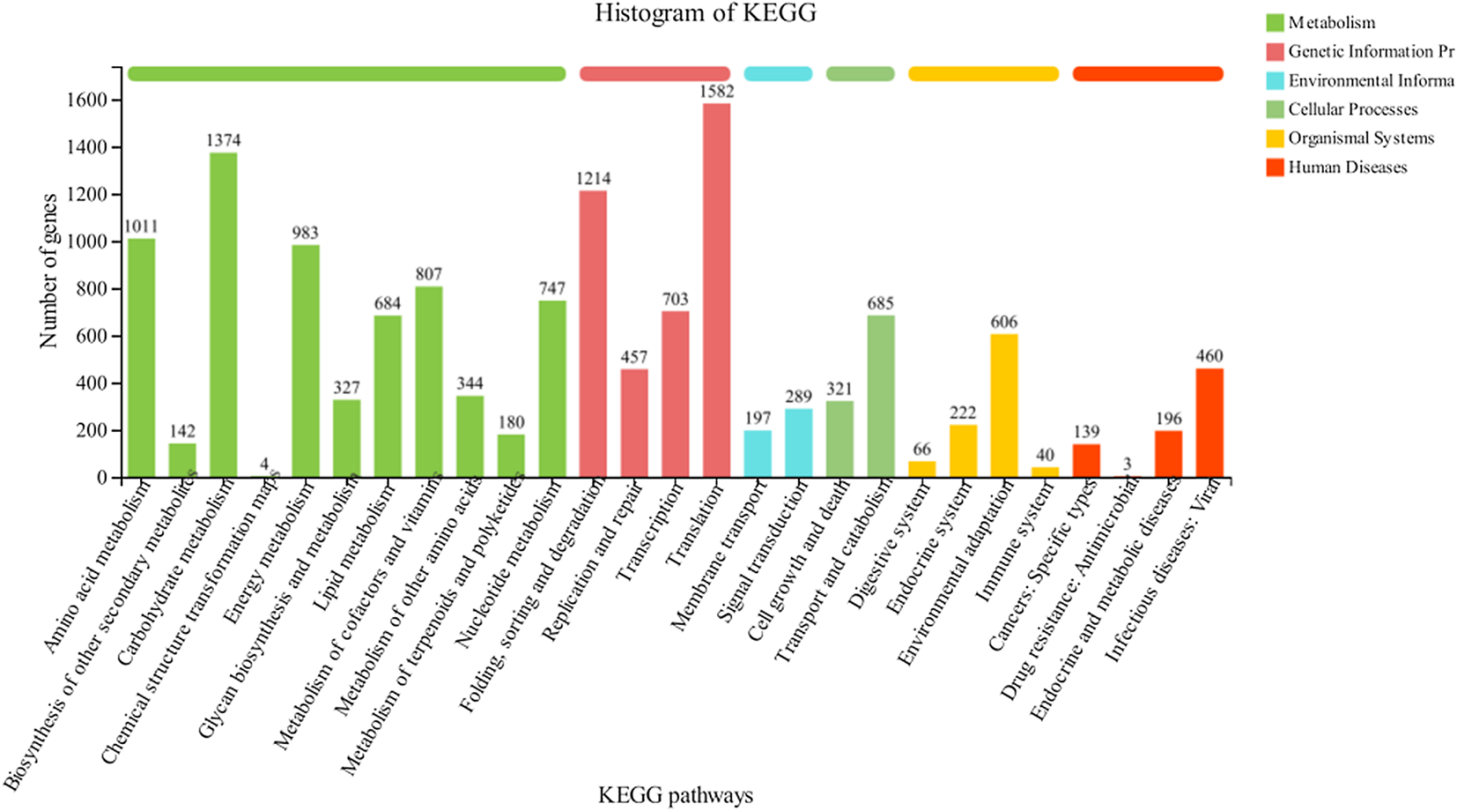
KEGG pathway classification of the unigenes. The *X* axis presents specific pathways in the second hierarchy. The different colors of the histogram represent six functional categories of pathways in the first hierarchy

Using the KEGG database, 16,796 unigenes were classified into pathways belong to six functional “first category”, consisting of “Metabolism”, “Genetic Information Processing”, “Environmental Information Processing”, “Cellular Processes”, “Organismal Systems”, and “Human Diseases”. The unigenes were classified into 27 pathways of “second category”, in which the largest number was 1,582 unigenes (11.48%) in the pathway of “Translation”, followed by 1,374 unigenes (9.97%) in the pathway of “Carbohydrate metabolism” and 1,214 unigenes (8.81%) in the pathway of “Folding, sorting and degradation” (Fig. 5). With high quality sequencing and assembly data (Tables 2, 3; Figs. 2, 3, 4, 5), the molecular processes/pathways involved in pressure response and adaptation of *H. pluvialis* were obtained. The results provided comprehensive information on genes/pathways involved in the determination of salicylic acid and high light response.

### Differentially expressed genes between treatment stages and KEGG pathway enrichments

Cluster analysis of the differentially expressed genes (DEGs) was conducted to judge the expression pattern of DEGs during different treatment stages. The cluster of DEGs revealed that SAHL_48 had significant different expression patterns with that of SAHL_24, SAHL_12, SAHL_6, SAHL_1 and Control. SAHL_24 was similar to SAHL_12 and SAHL_6, while SAHL_1 was more similar to Control, which means SAHL stresses caused pronounced changes in gene expression associated with treat time. To identify the DEGs correlating with SAHL, pairwise comparison was conducted among the six treatment stages. As shown in Table 4, changes in gene expression during the treatment stages were analyzed, and the number of the up- or down-regulated DEGs was also displayed. It was revealed that SAHL treatment stages from Control to SAHL_48 had the greatest DEGs number of 7,232 with 2,850 down-regulated and 4,381 up-regulated genes. While stage SAHL_24 relative to SAHL_48 had the fewest DEGs (2,030), in which 1,365 genes were significantly up-regulated and 665 genes were significantly down-regulated. Pair-wise comparison of DEGs between successive stages was evaluated to analysis the global gene expression changes during the treatment stages. Our results displayed that the number of the differentially expressed genes (DEGs) decreased according to sequential transition of these stages, and more DEGs were up-regulated for majority of the stage transitions (Table 4). It was indicated that more changes in transcriptional activity and functions were observed during immediately response of *H. pluvialis* to SAHL stresses during the first day of treatment, including the stage transitions from Control to SAHL_24. And, the adaption of *H. pluvialis* to SAHL stresses resulted in less changes in transcriptional activity and functions during stage transition from SAHL_24 to SAHL_48.

**Table 4:** Numbers of the DEGs which were involved in each treatment conditions

Stages	CN	SAHL_1	SAHL_6	SAHL_12	SAHL_24	SAHL_48
CN	–	1649/1044	2020/1256	2957/2190	3000/2464	4381/2850
SAHL_1	1044/1649	–	1447/1358	2548/2845	2538/2815	4113/3484
SAHL_6	1256/2020	1358/1447	–	1059/1162	1169/1220	2323/1537
SAHL_12	2190/2957	2845/2548	1162/1059	–	1395/1269	2613/1409
SAHL_24	2464/3000	2815/2538	1220/1169	1269/1395	–	1365/665
SAHL_48	2850/4381	3438/4113	1537/2323	1409/2613	665/1365	–

Biological pathways which were active under the SAHL stresses were identified by mapping the up-regulated and down-regulated DEGs to canonical signaling pathways obtained from annotation of the KEGG database (Additional file 1: Table S1). In the pairwise comparison of SAHL_1 vs. Control, 25 KEGG terms were significantly enriched (*p* < 0.05) by annotation of the 1,649 up-regulated DEGs, and none terms were significantly enriched by annotation of the down-regulated DEGs. Among them, numerous genes involved in chemical metabolism (e.g., “Glyoxylate and dicarboxylate metabolism”, “Nitrogen metabolism”, “Starch and sucrose metabolism”, “Alanine, aspartate and glutamate metabolism” and “Pyruvate metabolism”) and biosynthesis (e.g., “Carotenoid biosynthesis” and “Arginine biosynthesis”) were identified. Another large DEGs group was enriched in biological process, such as “ABC transporters”, “Oxidative phosphorylation”, “Arginine biosynthesis” and “Peroxisome”.

In the pairwise comparison of SAHL_6 vs. SAHL_1, 11 KEGG terms were significantly enriched (*p* < 0.05) by annotation of the 1,447 up-regulated DEGs, and 9 KEGG terms were significantly enriched (*p* < 0.05) by annotation of the 1,358 down-regulated DEGs. In which, the up-regulated DEGs were mainly related to photosynthesis (e.g., “Photosynthesis” and “Carbon fixation in photosynthetic organisms”) and metabolism (e.g., “Tyrosine metabolism” and “Phenylalanine metabolism”). While “Purine metabolism” and “ABC transporters” were the mainly involved pathways which were annotated by the down-regulated DEGs.

The 1,059 up-regulated DEGs in the pairwise comparison of SAHL_12 vs. SAHL_6 were significantly enriched in 15 KEGG terms, including “Glyoxylate and dicarboxylate metabolism”, “Pyruvate metabolism”, “Glycolysis/Gluconeogenesis”, “Purine metabolism”, “Carbon fixation in photosynthetic organisms”, “Pyrimidine metabolism” and so on. And, the 1,162 down-regulated genes in this transition stage were significantly enriched in 18 KEGG terms, consisting of “ABC transporters”, “Thermogenesis”, “Starch and sucrose metabolism” and “Phenylalanine metabolism” and so on.

In the pairwise comparison of SAHL_24 vs. SAHL_12, 18 KEGG terms were significantly enriched (*p* < 0.05) by annotation of the 1,395 up-regulated DEGs, which mainly involved in “ABC transporters”, “Thermogenesis”, “Starch and sucrose metabolism”, “Oxidative phosphorylation”, “Phenylalanine metabolism” and so on. And, there were 9 KEGG terms were significantly enriched by annotation of the 1,269 down-regulated DEGs, including “Ribosome biogenesis in eukaryotes”, “Purine metabolism”, “Glyoxylate and dicarboxylate metabolism”, “Alanine, aspartate and glutamate metabolism”, “Pyruvate metabolism” and so on.

For the pairwise comparison of SAHL_48 vs. SAHL_24, there were 8 KEGG terms which were significantly enriched by annotation of the 1,365 up-regulated genes. And, the main terms were related to “Endocytosis”, “Amino sugar and nucleotide sugar metabolism”, “Fructose and mannose metabolism” and “Photosynthesis”. The 665 down-regulated genes involved in this stage of transition were significantly enriched in 9 KEGG terms, such as “ABC transporters”, “Oxidative phosphorylation”, “Photosynthesis”, “Amino sugar and nucleotide sugar metabolism”, “Carbon fixation in photosynthetic organisms” and so on.

All the above results shown that SAHL stresses promoted the expression level of the up-regulated genes that related to KEGG pathways changing from chemical metabolism to ABC transporters, and then to Endocytosis according to treatment stages. And, the down-regulated expressed genes related to KEGG pathways of ABC transporters, Purine metabolism, Oxidative phosphorylation and Photosynthesis. Interestingly, KEGG pathways during stage transition of SAHL_1 to Control were all up-regulated and mainly associated with the biosynthesis of secondary metabolites. In general, KEGG enrichment analysis of the DEGs identified many transcripts that were associated with the biosynthesis of primary and secondary metabolites, photosynthesis, and immune system responses. These pathways were consistent with the roles of the stress resistance system in plants [5, 15–20]. The results indicated that the regulations subjected by *H. pluvialis* response to SAHL stresses changed along with the transition of the treatment stages.

### Gene expression profiling and KEGG pathway enrichments

**Fig. 6:**
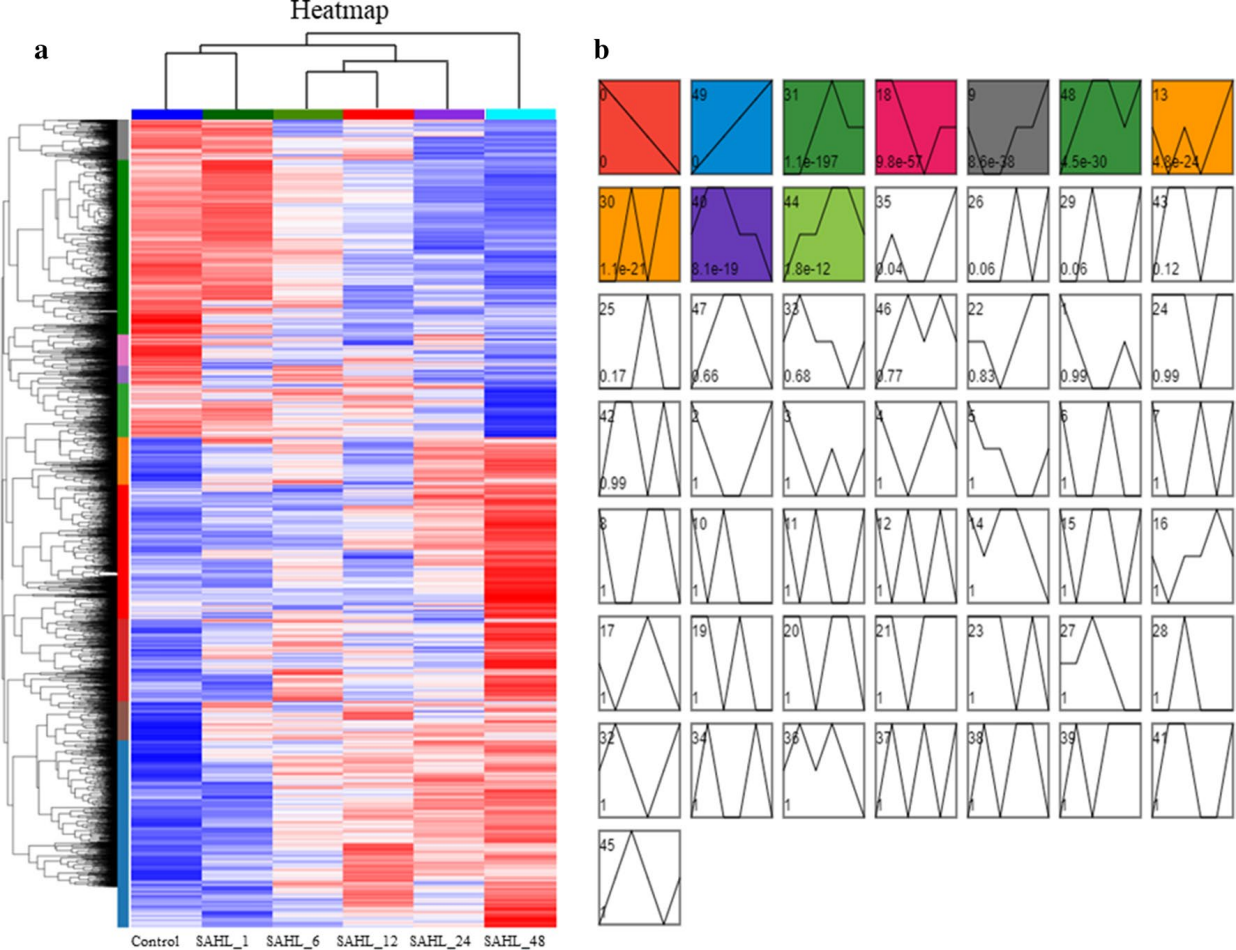
Cluster analysis and gene expression profiling of DEGs. **a** Cluster analysis of DEGs. The *X* axis presents treatments, while the left *Y* axis represents relative expression, the red and blue represent up-regulation and down-regulation. **b** Gene expression profiling of DEGs

In this study, 50 profiles were classified by gene expression pattern analysis throughout the treatment stages, and analysis with STEM revealed that 10 profiles, including profile 0, 49, 31, 18, 9, 48, 13, 30, 40 and 44 all had a *P*-value lower than 0.01 (Fig. 6).

The 2,045 genes included in profile 0 were down-regulated during the treatment stages from Control to SAHL_48 in a row. The significantly enriched (*p* < 0.01) KEGG pathways were related to “Phagosome”, “Relaxin signaling pathway”, “AGE-RAGE signaling pathway in diabetic complications”, “MAPK signaling pathway—plant” and “Human cytomegalovirus infection” (Additional file 1: Table S2). These pathways were mostly related to regulation of stress response and cellular proliferation and apoptotic regulation.

The expression pattern of the 1,895 genes in Profile 49 was up-regulated during the treatment stages from Control to SAHL_48 in contrary to that of Profile 0. In this profile, there were five significantly enriched KEGG pathways involving “DNA replication”, “Fatty acid degradation”, “Fatty acid elongation”, “Cutin, suberine and wax biosynthesis” and “Riboflavin metabolism”, which were mostly related to fatty acid biosynthesis and metabolism, and cell wall synthesis.

Profile 31 included 1,185 genes that were expressed at similar levels in treatment stage of Control and SAHL_1, and up-regulated during stage SAHL_1 to SAHL_12, and then down-regulated from stage SAHL_12 to SAHL_24, and expressed at similar levels in stage of SAHL_24 and SAHL_48. The significantly enriched pathways were “Ribosome biogenesis in eukaryotes”, “Citrate cycle (TCA cycle)”, “Purine metabolism”, “Alanine, aspartate and glutamate metabolism”, “Glyoxylate and dicarboxylate metabolism”, “Phenylalanine, tyrosine and tryptophan biosynthesis”, “Cysteine and methionine metabolism”, “Carbon fixation in photosynthetic organisms”, “Pyruvate metabolism”, “Isoquinoline alkaloid biosynthesis”, “Lysine biosynthesis” and “Tropane, piperidine and pyridine alkaloid biosynthesis”. These pathways were mainly related to primary metabolite metabolism and carbon fixation reaction.

Profile 18 had 769 genes that were lowest expressed during stage SAHL_12, and stage Control and SAHL_1 had the similar expression level which was the highest in this profile, and stage SAHL_24 and SAHL_48 had the similar expression level. The 3 significantly enriched pathways were “Glycerolipid metabolism”, “ABC transporters” and “Circadian rhythm—plant”.

Profile 9 consisted of 779 genes that were most highly expressed during stage SAHL_48 and lowest expressed during stage SAHL_1 and SAHL_6 but at similar levels at the other stages. None gene was significantly enriched (*p* < 0.01) in KEGG pathways in this profile.

The 872 genes in profile 48 had the lowest expression levels during stage Control, and the expression levels increased from stage Control to SAHL_6 to reach the highest level, and at similar levels among the stages of SAHL_12 and SAHL_48, while decreased during the stage SAHL_24. In this gene expression profile, the significantly enriched pathways were composed of “Nitrogen metabolism”, “Alanine, aspartate and glutamate metabolism”, “Fatty acid degradation”, “Tyrosine metabolism”, “Glycolysis/Gluconeogenesis”, “alpha-Linolenic acid metabolism”, “Indole alkaloid biosynthesis”, “Betalain biosynthesis” and “Isoquinoline alkaloid biosynthesis”. Hence, SAHL stresses might be accompanied by induced amino acid and glycometabolism. The up-regulation of amino acid and glycometabolism with treatment stages indicated massive targeted protein and glucose degradation occurred in *H. pluvialis* cells response to SAHL stresses.

The 744 genes involved in Profile 13 were lowest expressed during stage SAHL_1 and SAHL_12 and increased from stage SAHL_12 to SAHL_48 and reached the highest expression level. The significantly enriched pathways in this gene expression profile were “Fatty acid elongation”, “Cellular senescence” and “C-type lectin receptor signaling pathway”. The up-regulation of these pathways demonstrated cell aging and fatty acids accumulation occurred in *H. pluvialis* response to SAHL stresses.

The 631 genes in Profile 30 were highly expressed at stage SAHL_6, SAHL_24 and SAHL_48, and lowly expressed at the other stages. “Isoquinoline alkaloid biosynthesis”, “beta-Alanine metabolism”, “Tropane, piperidine and pyridine alkaloid biosynthesis”, “Phenylalanine metabolism” and “Tyrosine metabolism” were the significantly enriched pathways in this profile. These pathways were mainly related to amino acid metabolism and secondary metabolites biosynthesis.

There were 706 genes in Profile 40 which were highly expressed during stage SAHL_1 and SAHL_6, and lowest expressed during stage SAHL_48. Two significantly enriched pathways of this profile were “ABC transporters” and “Parathyroid hormone synthesis, secretion and action”. These pathways were mainly related to membrane transport and hormone biosynthesis.

Profile 44 consist of 667 genes which were highly expressed during stage SAHL_12 and SAHL_24, and lowest expressed at stage Control. There were 8 significantly enriched pathways in this profile, including “Glyoxylate and dicarboxylate metabolism”, “Citrate cycle (TCA cycle)”, “Indole alkaloid biosynthesis”, “Tryptophan metabolism”, “Photosynthesis—antenna proteins”, “Propanoate metabolism”, “Cysteine and methionine metabolism” and “Betalain biosynthesis”.

It was reported that high light is the direct factor for the changes in gene expression associated with many pathways in *H. pluvialis*, including photosynthesis-antenna proteins, carbon fixation in photosynthetic organisms, carotenoid biosynthesis, fatty acid elongation and so on [21]. Meanwhile, metabolomic changes in *H. pluvialis* upon exposure to high light stress were observed, which demonstrated a metabolic adaptive mechanism against high light stress [23]. Studies had indicated that more genes involved in the metabolism of carbohydrate, energy and amino acids were expressed in *H. pluvialis* cells once they were exposed to SA induction, and the results also revealed that the stress response of SA induction on *H. pluvialis* was more relevant to cell growth/death of cellular processing and genetic translation information [15]. In this study, the most significantly over-represented GO terms for DEGs were associated with biosynthetic process (Fig. 3). The top three significantly enriched KEGG pathways for DEGs were “Translation”, “Carbohydrate metabolism” and “Folding, sorting and degradation” (Fig. 5). According to the results revealed by the STEM analyses of the DEGs, the substantially up-regulated genes during transition of treatment stages were significantly enriched into functional pathways, including DNA replication, Fatty acid metabolism, Carbon fixation, Primary metabolic products and energy metabolism (Profile 49, 31, 48 and 13 in Additional file 1: Table S2). While the substantially down-regulated during treatment stages were significantly enriched into pathways, including phagosome, ABC transporters, Glycerolipid metabolism, signaling pathways and so on (Profile 0, 18 and 40 in Additional file 1: Table S2), which were mainly related to membrane transport and hormone biosynthesis. Summarily, results in this study were consistent with that of the previous reports that various metabolic processes and production of primary/secondary metabolites, e.g., carbohydrate, energy and amino acids, were regulated when *H. pluvialis* were upon exposure to SAHL stresses. Comparatively analysis of our results and the previous reports demonstrated that SA induced the up-regulation of genes involved in amino acids biosynthesis, primary metabolic products and energy metabolism, and genes relevant to cell growth/death of cellular processing and genetic translation information to promote self-protection process in *H. pluvialis*, leading to adaptation of *H. pluvialis* to high light stress.

### The transcription factors involved in salicylic acid and high light stresses

Transcription factors (TFs) are important regulatory proteins which bind to *cis*-regulatory specific DNA sequences, and these so-called *cis*-acting elements could enhance or repress RNA transcription of target genes [37, 38]. TFs have been recognized to play very important roles in plants to withstand unfavorable environmental conditions and control organ development [39, 40]. Lipid synthesis in animals, plants and microorganisms has been identified to be regulated by various TFs[37–41]. It was reported in a previous review that the overproduction of valuable metabolites could be acheived when stimulated by numerous TFs in different microalgae species [41]. For example, Gao et al. [15, 16] reported that the hormonal signal pathways of *H. pluviali* would be started by the TFs, which were induced by SA and JA. And, enhancing the enhanced biosynthesis of astaxanthin/carotene was thought to be regulated by the remarkable expression of MYB family in *H. pluviali* [15].

Based on the PlantTFDB database, the comparative transcriptome analysis of *H. pluvialis* among the treatment stages highlighted 705 unigenes as putative transcription factors (TFs) which were in response to SAHL stresses, and these unigenes were assigned to 25 families (Additional file 1: Table S3). The top five most abundant TFs families were C3H, MYB, Nin-like, MTB_related and ERF, which cover 53.26% of the putative TFs unigenes. According to differential numbers of the unique transcripts, the potential regulatory contributions of these TFs families might be characterized differently in astaxanthin and fatty acids biosynthesis pathways of this green microalga. For the presence of target gene signatures could be accurately predicted by TF regulation, the TFs with predicted target genes in connection with astaxanthin and fatty acids accumulation are listed in Table 5, and the RPKM values of these TFs at different treatment stages were also listed.

**Table 5:** Transcription factors involved in astaxanthin and fatty acid biosynthesis pathways and their expression level of FPKM in *H. pluvialis* in this study

Gene_id	TF family	Control	SAHL_1	SAHL_6	SAHL_12	SAHL_24	SAHL_48
Ch_GLEAN_10000740	CPP	10.64	26.76	24.7	24.57	24.07	33.20
Ch_GLEAN_10001490	bZIP	3.45	2.88	2.92	2.41	4.82	5.79
Ch_GLEAN_10003661	MYB_related	26.59	24.62	26.96	19.34	21.61	20.63
Ch_GLEAN_10004440	MYB	27.16	23.24	24.83	20.20	18.99	18.13
Ch_GLEAN_10004481	Nin-like	39.72	48.23	44.07	62.50	53.29	53.87
Ch_GLEAN_10005227	GATA	15.09	17.95	16.57	12.69	14.00	10.92
Ch_GLEAN_10011003	bHLH	52.21	65.50	60.51	42.37	46.51	43.70
Ch_GLEAN_10011496	CPP	11.36	14.55	14.01	14.84	15.14	16.43
Ch_GLEAN_10011915	bZIP	34.56	30.49	24.45	27.59	32.18	27.73
Ch_GLEAN_10012620	CPP	1.62	1.27	1.26	1.65	1.36	1.44

Based on the results obtained, C3H, MYB, Nin-like, MTB_related and ERF were the top five most highly expressed TF families. Ten putative TFs, which were assigned to 7 families, were characterized to participate in regulating the biosynthesis pathways of astaxanthin and fatty acid synthesis in *H. pluvialis*.

### Expression profiles of genes involved in carotenogenic and fatty acid biosynthesis pathways

**Fig. 7:**
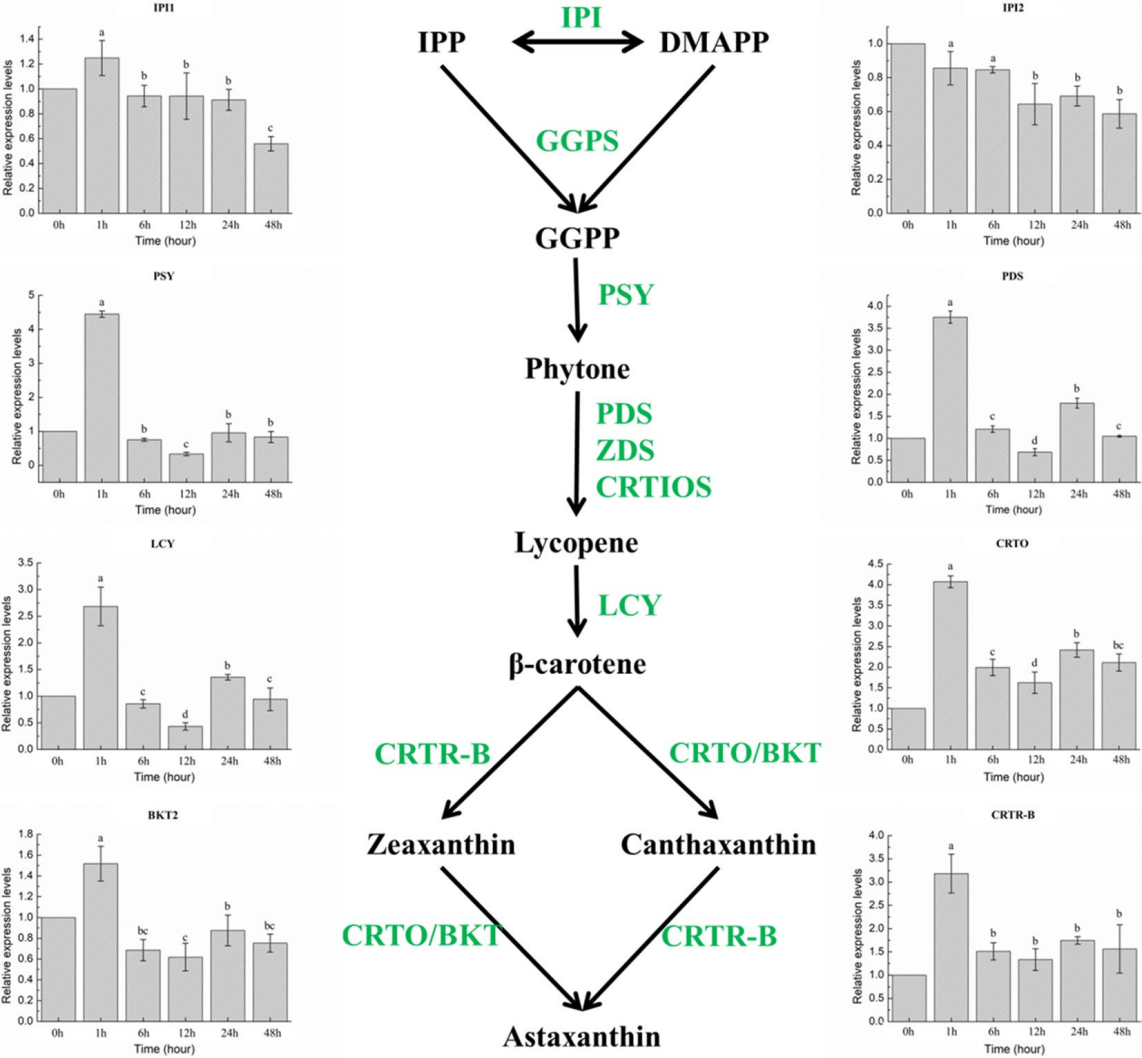
Expression of astaxanthin biosynthesis-related genes in *H. pluvialis* under different treatment stages. Note: Different superscript lowercase letters on top of the columns indicate significant differences (*p* < 0.05) among the treatments, and any of the same lowercase letters or letter said the difference was not significant (*p* > 0.05). The following figure is the same

**Fig. 8:**
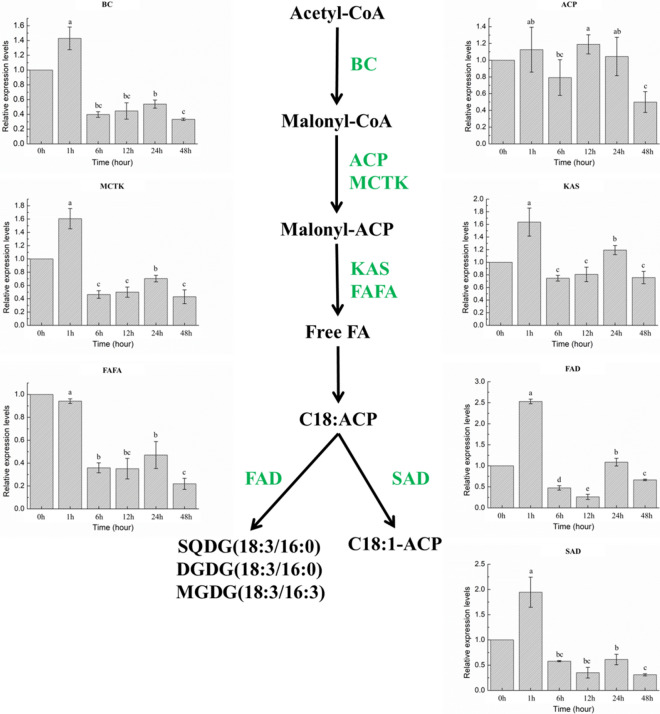
Expression of genes in *H. pluvialis* fatty acid biosynthesis pathway under different treatment stages

In this study, both expressions of key carotenogenic and fatty acid biosynthesis genes were determined under different treatment stages. The expression level of these genes varied among the stages (Figs. 7, 8). Pearson Correlation analysis (SPSS19.0) was carried out to study the relationship among the gene expressions, and the specific results are displayed in Table 6.

**Table 6:** Correlations between expression of carotenogenic and fatty acid biosynthesis genes (cofactors, Pearson Correlation in SPSS19.0)

Genes	IPI1	IPI2	PDS	PSY	LCY	CRTO	CRTR_B	BKT	BC	ACP	MCTK	KAS	FAFA	FAD	SAD
IPI1	1	0.612^**^	0.612^**^	0.609^**^	0.502^*^	0.404	0.503^*^	0.528^*^	0.726^**^	0.554^*^	0.726^**^	0.598^**^	0.685^**^	0.609^**^	0.719^**^
IPI2	0.612^**^	1	0.230	0.281	0.255	-0.080	0.105	0.379	0.591^**^	0.262	0.526^*^	0.252	0.712^**^	0.348	0.446
PDS	0.612^**^	0.230	1	0.958^**^	0.963^**^	0.917^**^	0.905^**^	0.852^**^	0.769^**^	0.250	0.838^**^	0.896^**^	0.529^*^	0.952^**^	0.860^**^
PSY	0.609^**^	0.281	0.958^**^	1	0.937^**^	0.861^**^	0.877^**^	0.888^**^	0.848^**^	0.229	0.892^**^	0.852^**^	0.614^**^	0.957^**^	0.919^**^
LCY	0.502^*^	0.255	0.963^**^	0.937^**^	1	0.861^**^	0.833^**^	0.897^**^	0.791^**^	0.191	0.821^**^	0.929^**^	0.584^*^	0.961^**^	0.866^**^
CRTO	0.404	-0.080	0.917^**^	0.861^**^	0.861^**^	1	0.935^**^	0.643^**^	0.519^*^	0.123	0.610^**^	0.770^**^	0.209	0.800^**^	0.675^**^
CRTR_B	0.503^*^	0.105	0.905^**^	0.877^**^	0.833^**^	0.935^**^	1	0.680^**^	0.635^**^	0.156	0.720^**^	0.730^**^	0.318	0.819^**^	0.694^**^
BKT	0.528^*^	0.379	0.852^**^	0.888^**^	0.897^**^	0.643^**^	0.680^**^	1	0.876^**^	0.318	0.897^**^	0.855^**^	0.736^**^	0.929^**^	0.895^**^
BC	0.726^**^	0.591^**^	0.769^**^	0.848^**^	0.791^**^	0.519^*^	0.635^**^	0.876^**^	1	0.360	0.967^**^	0.805^**^	0.892^**^	0.886^**^	0.907^**^
ACP	0.554^*^	0.262	0.250	0.229	0.191	0.123	0.156	0.318	0.360	1	0.414	0.455	0.413	0.269	0.382
MCTK	0.726^**^	0.526^*^	0.838^**^	0.892^**^	0.821^**^	0.610^**^	0.720^**^	0.897^**^	0.967^**^	0.414	1	0.827^**^	0.843^**^	0.921^**^	0.923^**^
KAS	0.598^**^	0.252	0.896^**^	0.852^**^	0.929^**^	0.770^**^	0.730^**^	0.855^**^	0.805^**^	0.455	0.827^**^	1	0.659^**^	0.921^**^	0.854^**^
FAFA	0.685^**^	0.712^**^	0.529^*^	0.614^**^	0.584^*^	0.209	0.318	0.736^**^	0.892^**^	0.413	0.843^**^	0.659^**^	1	0.686^**^	0.823^**^
FAD	0.609^**^	0.348	0.952^**^	0.957^**^	0.961^**^	0.800^**^	0.819^**^	0.929^**^	0.886^**^	0.269	0.921^**^	0.921^**^	0.686^**^	1	0.909^**^
SAD	0.719^**^	0.446	0.860^**^	0.919^**^	0.866^**^	0.675^**^	0.694^**^	0.895^**^	0.907^**^	0.382	0.923^**^	0.854^**^	0.823^**^	0.909^**^	1

*. Indicated that there was a significant correlation between the expression genes at *p* < 0.05 level; **. Indicated that there was a significant correlation between the expression genes at *p* < 0.01 level

### Transcript expression of carotenogenic genes

As is well known, isopentenyl pyrophosphate isomerase (IPI) [42], phytoene synthase (PSY) [43], phytoene desaturase (PDS) [44–47], lycopene β-cyclase (LCY) [3, 4, 22, 47], carotenoid oxygenase (CRTO), carotenoid hydroxylase (BKT2) and β-carotene hydroxylase (CRTR-B) are important carotenoid biosynthesis enzymes in *H. pluvialis* [11, 12, 14], in which PSY and PDS are the rate-limiting steps of the carotenoid biosynthesis pathway [43–47].

It has been reported by Gwak et al. [6] that the genes involved in the carotenoid biosynthesis pathway of *H. pluvialis*, including BKT, PSY and PDS, were all up-regulated upon exposure to high irradiance stress by transcriptome analysis. Gao et al. [15] revealed that the expression pattern of five genes (including ZDS, PDS, CRTZ, CRTB and ZEP) were different between jasmonic acid and salicylic acid inductions. Up-regulation of the PSY, PDS, ZDS and CRTR-B genes were correlated to excessive astaxanthin accumulation in *H. pluvialis*, but the patterns were different along with the same level of jasmonic acid or salicylic acid inductions. Transcriptome analysis was also used by He et al. [21] to conduct a synergistic research of three factors—high light irradiation, acetate and Fe^2+^—on the molecular mechanism of astaxanthin accumulation in *H. pluvialis* at the red-cell stage. It was demonstrated that the expression of more genes in the carotenoid biosynthesis pathway were identified under high light stress than the other two stress conditions, including PDS, CRTISO, LCYB, LUT1, LUT5 and ZEP[21]. Expression level of beta-carotene hydroxylase (CRTZ) was affected by high light irradiation and was observed significantly increased under acetate[21]. Meanwhile, astaxanthin accumulation was further enhanced by the over-expression of CRTZ and inhibited expression of LCYE[21]. It was revealed that the main driver of changes in gene expression involved in carotenoid biosynthesis is high light irradiation [21]. Zhao et al. [5] systematically examined differential gene expressions of *H. pluvialis* responding to nitrogen starvation, and foldings of the differentially expressed genes involved in carbon fixation pathway for astaxanthin biosynthesis and lipid metabolism had been comprehensively compared based on the mode of action upon exposure to nitrogen starvation. It was displayed that the expression of astaxanthin biosynthesis relating genes were significantly enhanced, and astaxanthin was accumulated by disposing carbon through MEP pathway to defense the stress. In particularly, the expression levels of the IPI, PSY, ZDS, CHYB and BKT genes increased more than fivefold.

In this study, the expression level of the selected carotenogenic genes, involving IPI, PSY, PDS, LCY, CRTO, CRTR-B and BKT, were varied among the treatment stages (Fig. 7). In detail, IPI1 was over-expressed at transcriptional level on stage SAHL_1 (*p* < 0.05), and then back to primary level (Control) during the rest stages. Differently, IPI2 expression was continuously inhibited followed by the extension of SAHL treatment. The expression of PSY, PDS, LCY, CRTO, BKT and CRTR-B genes displayed a similar pattern to each other. This expression pattern indicated that these genes were over-expressed at stage SAHL_1, and down-regulated during treatment stage from SAHL_1 to SAHL_12, and then up-regulated from stage SAHL_12 to SAHL_48. And, the expression level at stage SAHL_24 was higher than that during stage SAHL_48. All the genes involved in astaxanthin biosynthesis pathway revealed the same expression pattern, which was immediately up-regulated from Control to stage SAHL_1 and then displayed a decreasing trend from treatment stage of SAHL1 to SAHL_12, and increased from stage SAHL_12 to SAHL_48. The expression pattern of majority of these genes (including IPI2, PSY, PDS, LCY, CRTO, CRTR-B and BKT) showed a similar result with transcriptome analysis.

### Transcript expression of fatty acid biosynthesis genes

Biotin carboxylase (BC), Acyl carrier protein (ACP), Malonyl-CoA: ACP transacylase (MCTK), 3-ketoacyl-ACP synthase (KAS), Acyl-ACP thioesterase (FATA), Stearoyl-ACP-desaturase (SAD) and ω-3 fatty acid desaturase (FAD) are fatty acid synthesis enzymes, in which MCTK catalyzes the length extension of the growing acyl chain in the fatty acid elongation step through transfer malonyl moiety from malonyl-CoA onto the acyl carrier protein. After that, condensation reaction between acetyl CoA and malonyl ACP is catalyzed by KAS. And, hydrolysis of the thioester bond of acyl-ACP is catalyzed by the chain-length-determining enzyme FAFA to release free fatty acid and ACP. SAD is an important enzyme to catalyze the conversion of 18:0 to C18:1n9 which will determine the ratio of saturated to unsaturated fatty acids, and conversion of C18:2n6 to C18:3n3 is catalyzed by FAD [16, 27, 48].

It is widely recognized that astaxanthin accumulation in *H. pluvialis* is correlated with fatty acid synthesis upon exposure to stress conditions [50, 51]. The accumulation of storage lipid in *H. pluvialis* cells was observed to be substantially stimulated by high light irradiance stress, which was regulated by expression of de novo fatty acid biosynthesis-related genes at the transcription level [6]. It was also reported by He et al. [21] that the fatty acid biosynthesis relating related genes, especially the genes that involved in the fatty acid elongation pathway, were significantly affected by the high light irradiation. Expression of the fatty acid biosynthesis relating genes—KCS and MECR—were further enhanced upon exposure to addition of acetate. A similar effect of high light and acetate on expression levels of both carotenoid and fatty acid biosynthesis genes was observed. However, none direct effect of Fe^2+^ on the astaxanthin biosynthesis relating related genes in *H. pluvialis* was revealed. But those genes were indirectly promoted by oxidative stress, affecting the photosynthesis relating genes and thereby promoting astaxanthin biosynthesis. It was displayed by Zhao et al. [5] that all the transcripts related to fatty acids biosynthesis showed a higher expression level with nitrogen starvation.

These genes have been studied in this study as well, and it was revealed that the mRNA expressions of the most genes, including BC, ACP, MCTK, KAS, FAD, and SAD, were up-regulated at stage SAHL_1, and down-regulated according to time extension of the treatment stage from SAHL_1 to SAHL_12, and then up-regulated from stage SAHL_12 to SAHL_24, and then decreased again during stage SAHL_48. FAFA was down-regulated according to time extension under SAHL stresses (Fig. 8). Based on the results obtained, expression pattern of majority of the fatty acid biosynthesis relating genes displayed the same. Expression pattern of these fatty acid biosynthesis relating genes were consistent with that of the related genes in astaxanthin biosynthesis pathway. Take the significantly decreased cell density into consideration, fatty acids accumulated in individual cells were observed increasing with time course of SAHL treatment (Table 1).

According to the results reported by Hu et al. [23], ROS burst and photo-inhibition in *H. pluvialis* were observed at the beginning of high light stress. *H. pluvialis* underwent dramatic transcriptional changes to provide an adaptive mechanism against the high light stress, and over-produced astaxanthin through up-regulation of almost all the genes involved in MEP and astaxanthin biosynthesis pathway and down-regulation of those in the branch pathways. SA can regulate various plant metabolic processes and modulate the production of varied secondary metabolites, to protect plants against abiotic stress conditions [10]. Investigation of salicylic acid (SA) on the antioxidant system in *H. pluvialis* displayed that secondary carotenogenesis was affected by SA mainly through scavenging the free radicals, which was necessary for the induction of secondary carotenoid [2]. Enhanced accumulation of astaxanthin in *H. pluvialis* was considered to be part of the self-protection process of the cells in stress environments [6]. It was previously reported that the carotenogenic genes were responsible to supply the different units of astaxanthin biosynthesis under hormone stress, e.g., salicylic acid (SA) and jasmonic acid (JA), and the rapidly accumulation of astaxanthin was induced by the up-regulation of these genes [15, 16]. The transcriptional expression levels of FA biosynthesis genes were down-regulated in the first 6 h, and then up-regulated at 24 h in *H. pluvialis* upon induction of SA, and the up-regulation of FAD was regarded to be part of the self-protection mechanism in *H. pluvialis* by enhanced accumulation of C18:3n-3 FAs [16]. In this study, majority of the genes involved in fatty acid and carotenoid biosynthesis pathways showed the similar expression pattern, resulting in accumulation of astaxanthin and fatty acids in *H. pluvialis* cells. Thus the up-regulation of the key genes involved in fatty acid and carotenoid biosynthesis pathways in the first 1 h were speculated to stimulate the related metabolites to defense against the high light stress. Then, the down-regulation of these genes in 2 day cultivation might due to *H. pluvialis* had developed metabolic processes to facilitate their adaptation ability by counteracting adverse effects of excess ROS induced by high light stress, e.g., primary/secondary metabolic products, energy metabolism, immune system responses and cell growth/death of cellular processes, which were regulated by SA. The up- and down-regulations of these gene transcript levels might highlight the potential effect of multigene-encoded complex or gene post-translational modifications involved in astaxanthin and fatty acid accumulation within the cells. SA might play a role as a signaling molecule in self-protection processes in cells, leading to adaption of *H. pluvialis* to high light stress.

### Correlations between expression of carotenogenic and fatty acid biosynthesis genes

Expressions of the key carotenogenic and fatty acid biosynthesis genes during different SAHL treatment stages were determined in this study. To study the relationship between these gene expressions, Pearson Correlation (PC) analysis (SPSS19.0) was carried out and the results are summarized in Table 6.

According to the results in Table 6, there existed differences in the correlations between the genes. In general, none of the gene expressions was observed negatively correlated with other genes in this study. The expression of IPI1 shared close correlations with that of the carotenogenic genes analysed in this study except CRTO gene, while it was significantly correlated with all genes involved in fatty acid biosynthesis pathway. IPI2 was perceived not significantly correlated with the other genes involved in carotenoid biosynthesis pathway except IPI1 gene, but it had significant correlation with several genes involved in fatty acid biosynthesis pathway, including BC, MCTK and FAFA genes. CRTO gene expression was found to have closely relationships with PSY, PDS, LCY, CRTR_B, BKT, BC, MCTK, KAS, FAD and SAD gene expressions. ACP gene in fatty acid biosynthesis pathway was observed significantly correlated with IPI1 only, which were involved in the pathway of carotenoid biosynthesis in this study. Finally, almost all the other genes that were involved in both carotenoid biosynthesis and fatty acid biosynthesis pathways, including PSY, PDS, LCY, CRTO, CRTR-B, BKT, BC, MCTK, KAS, FAD and SAD, had significant or very significant correlation with one another.

Results obtained in the present PC analysis demonstrated that the gene clusters involved in carotenoid and fatty acid biosynthesis pathways had a significant positive correlation with each other, indicating that these two pathways were stoichiometrically coordinated at gene level. At the same time, ACP and IPI1 gene might play an important role in this coordinate relationship. Our detailed analysis of correlations between genes involved in astaxanthin and fatty acid biosynthesis pathways provided some interesting hints for molecular mechanisms of the coordination between these two pathways (Table 6).

### Predicted Protein–Protein Interaction Networks of carotenogenic and fatty acid biosynthesis genes

In this study, protein–protein interactions (PPI) networks of the carotenogenic and fatty acid biosynthesis genes were predicted using the web-tool STRING 10. Protein datasets of *H. pluvialis* based on transcriptome in this study were constructed. The protein homologs of carotenogenic and fatty acid biosynthesis genes in *Chlamydomonas reinhardtii* were analyzed by sequence BLAST based on the TAIR database, and then the proteome-scale interaction network was created by subjecting the homologs to the molecular interaction tool of STRING 10 [49].

**Fig. 9:**
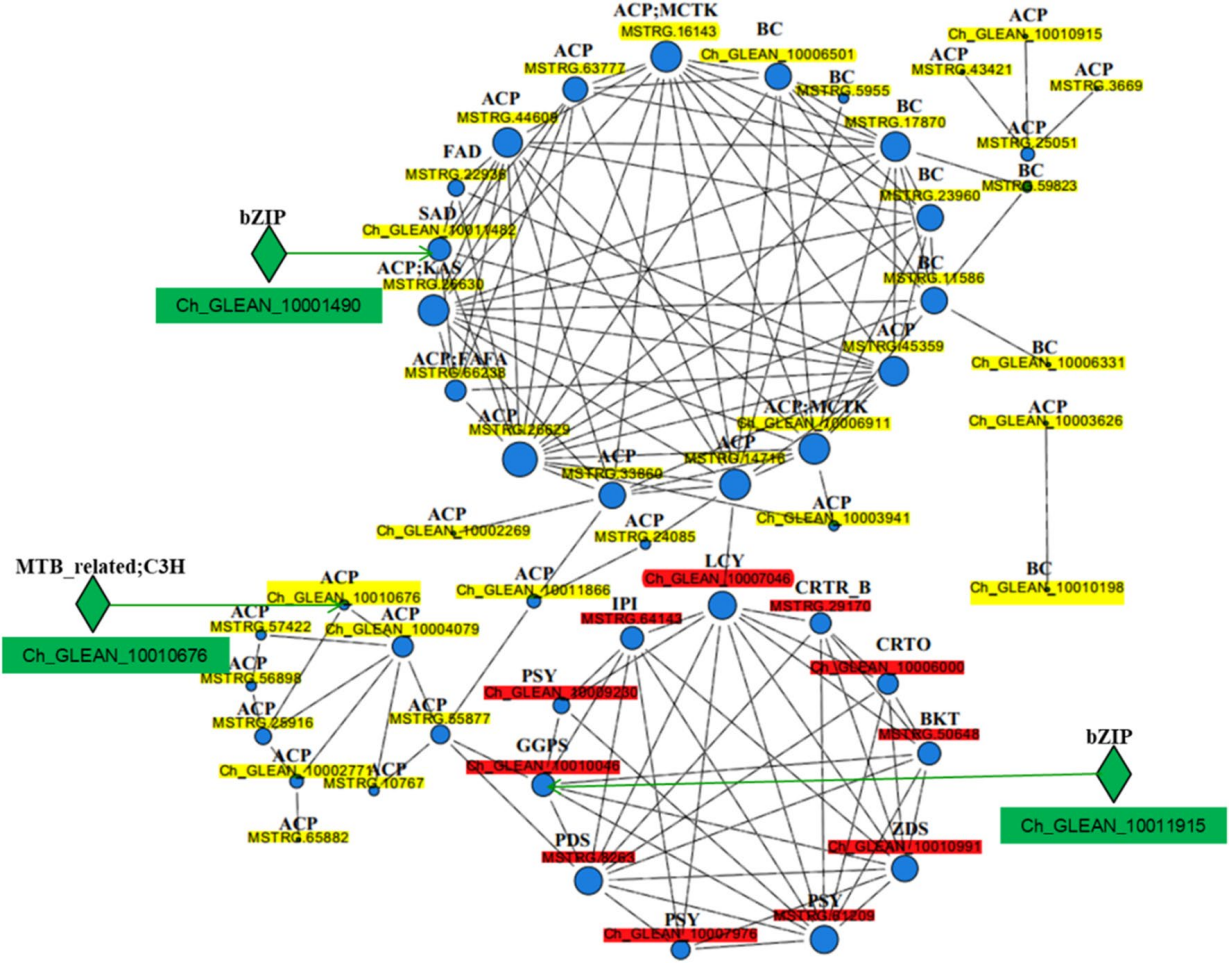
Protein–protein interaction (PPI) network in *H. pluvialis* based on carotenogenic and fatty acid biosynthesis genes. Three main groups were indicated in different colors, in which gene IDs with yellow, red and green background were genes involved in fatty acids biosynthesis, carotenoid biosynthesis modules and putative TFs, respectively

A total of 232 genes with carotenoid and fatty acid biosynthesis functions were selected for analysis (Additional file 1: Table S4). Then the web-tool STRING 10 was used to predict PPI based on these genes, and the proteome-scale interaction network is shown in Fig. 9. Among them, 53 genes were depicted in the STRING database, and two functional modules were illuminated in the network. Each of the functional modules was tightly-connected clusters, in which thicker lines represented stronger associations between the genes. The relationship of proteins in each module indicated that carotenoid and fatty acid biosyntheses were at a reasonable degree of independence, and there existed interactions between the proteins that were illuminated in these two modules. For example, MSTRG.14716 involved in fatty acid biosynthesis module was directly related to Ch_GLEAN_10007046, which was carotenoid biosynthesis functional involved. MSTRG.33860 and MSTRG.14716 involved in fatty acids biosynthesis module were connected with Ch_GLEAN_10010046 and MSTRG.8263 in carotenoid biosynthesis module through MSTRG.55877 and Ch_GLEAN_10011866. Additionally, three genes involved in carotenoid and fatty acid biosynthesis functions, including Ch_GLEAN_10011482, Ch_GLEAN_10010676 and Ch_GLEAN_10010046, were target genes of three putative TFs in response to SAHL stresses. These putative TFs were composed of Ch_GLEAN_10001490, Ch_GLEAN_10003661 and Ch_GLEAN_10011915, which were assigned to bZIP, MYB_related and CH3 families. According to the results obtained, PPI network analysis (Fig. 10) showed a similar result with that of PC analysis, indicating that functional modules involved in carotenoid and fatty acid biosynthesis were tightly-connected at a reasonable degree of independence. The interactions between these two modules were mainly between ACP, IPI, LCY, GGPS and PDS genes, and it was revealed that ACP gene might play an important role in coordinate relation of astaxanthin and fatty acid biosynthesis in *H. pluvialis*.

In general, the expression pattern of astaxanthin and fatty acid biosynthesis genes according to the extension of treatment time was consistent with a previous study reported by Ma et al. [27], which displayed that astaxanthin and fatty acids accumulation were induced under nitrogen starvation, along with over-expression of associated genes (except for IPI1 and IPI2). However, our results were different from that reported by Hu et al. [51], which eliminated possible molecular mechanisms of the coordination between astaxanthin and fatty acid biosynthesis in *H. pluvialis* was inter-dependence at the transcriptional level, and considered this interaction as feedback-coordinated at the metabolite level. Moreover, TFs might play roles in astaxanthin and fatty acid biosynthesis in this green microalga at the pre-transcriptional level.

## Conclusions

In this study, *Haematococcus pluvialis* showed enhanced accumulation of astaxanthin and fatty acids in single cells upon exposure to salicylic acid and high light (SAHL) stresses during the 2 day cultivation. Comparative transcriptomic analysis identified a large number of DEGs associated with the biosynthesis of primary and secondary metabolites, energy metabolism, photosynthesis, and immune system responses in response to SAHL stresses. The results indicated that high light stress directly up-regulated KEGG pathways in *H. pluvialis*, including carotenoid biosynthesis, fatty acid biosynthesis and carbon fixation. Exogenous SA provided adaptive mechanism for this microalga via the changed expression of genes involved in the metabolism of primary metabolites, energy and amino acids, and genes relevant to cell growth/death of cellular processing and genetic translation information. It is hypothesized that exogenous SA might play a role as a signaling molecule in self-protection processes in cells through altering the cell’s bioprocesses and immune system responses to tolerant photooxidative stresses, resulting in adaptation of *H. pluvialis* to high light stress. Genes encoding key enzymes involved in the astaxanthin and fatty acid biosynthesis pathways were studied, and majority of them displayed the similar expression pattern, which was up-regulated immediately upon exposure to SAHL stresses and then down-regulated with time course and then fluctuated. Pearson Correlation (PC) and protein–protein interactions (PPI) network analysis of these carotenogenic and fatty acid biosynthesis genes demonstrated that coordination between astaxanthin and fatty acid biosynthesis might be at transcriptional level. Therefore, it is necessary to perform an in-depth functional analysis of coordination between the important genes associated with astaxanthin and fatty acid biosynthesis. A number of transcription factors were identified in this study, and it was revealed that TFs might play roles in astaxanthin and fatty acid biosynthesis in this green microalga at the pre-transcriptional level. Besides, the function of TFs is needed to be studied to further investigate their potential roles in stress responses of *H. pluvialis*, coupled with astaxanthin and fatty acids production. With regards to better astaxanthin and fatty acids induction efficiency in the SA treatment [3, 4, 15, 16], this study provides new insight to effects of SA treatment on fatty acids and astaxanthin biosynthesis in response to high light stress. These results also form a basis to facilitate future research on a genetic bioengineer approach for large-scale production of astaxanthin and fatty acids in *H. pluvialis*.

## Materials and methods

### Strains and culturing conditions

In this study, *H. pluvialis* 192.80 obtained from Sammlung von Algenkulturen Culture Collection of Algae at Gottingen University was cultured in BBM (Bold Basal Medium) media in 250-mL Erlenmeyer flasks with permeable sealing membrane. Cells were kept at 22 °C under continuous fluorescent light (20 μmol·m^−2^·s^−1^) until their growth reached the logarithmic phase (about 1 × 10^5^ cells·mL^−1^). Then the alga cultures were collected together and evenly divided into 18 aliquots of 300 mL each in 500 mL Erlenmeyer flasks, which were used for salicylic acid (SA) and high light (HL, 350 μmol·m^−2^·s^−1^) treatments (SAHL). Each treatment was comprised of three biological repeats. The SA dosages were at 25 mg·L^−1^ for the induction, which were displayed by the previous reports that this dosages had an optimal effect on *H. pluvialis* [4, 15, 16]. The cultures were sampled at 0, 1, 6, 12, 24 and 48 h, which were represented as Control, SAHL_1, SAHL_6, SAHL_12, SAHL_24 and SAHL_48, respectively. Samples were centrifuge-harvested at 6000 g for 10 min, immediately frozen in liquid nitrogen, and stored at − 80 °C until further analysis.

### Microscopy observation of microalgal morphology

On each sampling time, the alga cells were observed using an Olympus BX61 microscope and an Olympus DP10 digital camera, and the progress in cell morphology and colour changes of *H. pluvialis* in the treatments were tracked.

### Growth, lipid and pigments analyses

As a quantitative way, cell number was calculated to measure the growth rate of *H. pluvialis*. Dry cell weight (DCW) was determined to measure the biomass accumulation of *H. pluvialis* for each treatment. In this study, cell numbers were calculated by Thoma counting method using an Olympos CX40 microscope and the biomass DCW was measured gravimetrically on every sampling time [29]. Pigment content was determined using a spectrophotometric method, and carotenoid concentrations were determined according to the method reported by Cheng et al. [30]. Fatty acid methyl ester (FAME) analysis was determined via the method previously optimized by Ma et al. [27] and astaxanthin yield was measured via spectrophotometric method [31].

### RNA extraction, library construction and sequencing

Total RNA was extracted using Trizol reagent (Invitrogen, CA, USA) in accordance with the manufacturer’s protocol. The quality of the extracted RNA was checked by agarose gel electrophoresis and the BioPhotometer Plus photometer (Eppendorf, Germany). The Agilent 2100 Bioanalyzer (Agilent Technologies, USA) was used to evaluate RNA integrity, and samples with RNA integrity number ≥ 5 were subjected to the subsequent analysis. Then libraries were constructed using Illumina TruseqTM RNA sample prep Kit (Illumina, CA, USA) following the manufacturer’s instructions. Purification was conducted using Oligo dT beads (ThermoFisher, German), and PCR reaction were used to amplify the purified samples. Then Agilent 2100 Bioanalyzer were used to validate libraries of these purified samples, and qualified libraries were sequenced on the Illumina sequencing platform (HiSeq™ 2500) using the paired-end technology (PE150) by Majorbio Co. (Shanghai, China, http://www.majorbio.com/). Finally, 150 bp/150 bp paired-end reads were generated.

### Sequence assembly and annotation

Raw reads were processed using FastX—Toolkit, and reads containing ploy-N and the low-quality reads were removed by SeqPrep and Sickle to obtain clean reads, which were then mapped to the reference genome using TopHat2. The TopHat-Cufflinks platform were used to reassemble the mapped reads, and Cuffcompare software was used to perform gene structure extension and novel transcript identification through comparation of reference genome and known annotated genes. Following the assembly, Blast + was used to compare these unigenes with the non-redundant database, and the cut-off E-value of < 10^–5^ was selected.

The assembled sequences were annotated with several protein databases, including Gene Ontology (GO), Kyoto Encyclopedia of Genes and Genomes (KEGG) and Cluster Orthology Genome (COG). Cufflinks and HTSeq package in 2010 were used to calculate the Fragments Per Kilobases per Millionreads (FPKM) value and read counts of each gene, respectively. The differentially expressed genes (DEGs) were identified using the DEGSeq, with p-adjust < 0.001 and |log2FC|> = 1 setting as the threshold to indicate significant differential expression.

Hierarchical cluster analysis was performed to explore gene expression patterns of the DEGs. For the up- and down-regulated DEGs observed between the treatment stages, GO and KEGG enrichment analyses were performed and the most significantly enriched biological processes and terms were highlighted. Gene expression trends were analyzed and clustered using the software Short Time-series Expression Miner (STEM), and the genes were clustered into 50 expression profiles. Profiles with *p* < 0.01 were then separately subjected to KEGG database for pathway enrichment, and the significantly enriched pathways with *p* < 0.01 were focused. Fast prcomp functions (Molecular Devices, LLC, CA, US) were used to perform principal component analysis of the six gene libraries, and probe-sets of the best fit the first principal component (PC1) and PC2 were selected using score matrix. Based on the COG data, putative transcription factors (TFs) were identified by searching the Plant Transcription Factor Database (PlantTFDB 4.0). The Search Tool for the Retrieval of Interacting Genes/Proteins (STRING) algorithm was employed to analyses the network of functional interaction among selected genes. All these analyses mentioned above were performed based on the integrated cloud platform of I-Sanger (https://www.i-sanger.com/), and the methodology on data analysis was presented as a work flow, which was provided in Additional file 1: Fig. S2.

### Real-time quantitative reverse transcriptase PCR

To investigate the expression profiles of sixteen genes including eight kinds of carotenoid synthesis genes, seven kinds of fatty acid synthesis genes, and the internal control gene (β-actin) simultaneously in response to SAHL stresses, the real-time fluorescence quantitative PCR (qRT-PCR) was performed. Firstly, RNA was isolated from the time-course samples using a RNA fast 200 kit (Fastagen, Shanghai, China) according to the manufacturer’s protocol. Secondly, first-strand complementary DNA (cDNA) synthesis was performed using a PrimeScript^TM^RT reagent Kit with gDNA Eraser (Perfect Real Time) (TaKaRa). Thirdly, oligo dT primers were used to synthesize a total of 0.5 μg RNA in a 20 μL reaction to cDNA for further qRT-PCR analysis. Finally, qRT-PCR was carried out using pairs of gene-specific primers listed in Additional file 1: Table S5, which were designed according to GenBank data as previously reported by Gao et al. [4], Lei et al. [33] and Ma et al. [27]. Expression levels were normalized using the Actin transcript level, as it has been used in other studies [27, 32, 33]. The qRT-PCR was performed with four independent biological and two technical replicates on a ABI QuantStudio™ 6 Flex System (Applied Biosystems, USA) following the protocol previously described [27] using a SYBR Green based PCR assay. LinRegPCR program was employed to determine the PCR efficiency for each sample, and primer efficiency (PE) was calculated by the mean of efficiency values obtained from the individual samples [34, 35]. Expression levels of tested genes were determined and calculated by 2^−ΔΔCt^ methods [36].

### Statistical analysis

Experiments were conducted with biological triplicates from the separate microalgal cultures in this study except the transcriptome analysis. Data in the figures and tables were shown as the average of triplicates with standard errors. One-way ANOVA (SPSS19.0) was performed for statistically analysis, and *P*-values of < 0.05 were considered as statistically significant.

## Supplementary Information

**Additional file 1. **Additional figures and tables.

## Data Availability

The authors promise that all data generated or analyzed in the present study are included in this article and in additional information.
