# Supplementary material for: Transcriptome-based analysis of the effects of salicylic acid and high light on lipid and astaxanthin accumulation in Haematococcus pluvialis
Source: Biotechnol Biofuels. 2021 Apr 1;14:82. doi: 10.1186/s13068-021-01933-x (PMC8017637; doi:10.1186/s13068-021-01933-x)
Supplement: Supplementary file 1 — Additional file 1. Additional figures and tables. [file 13068_2021_1933_MOESM1_ESM.docx]

**Supplement material**


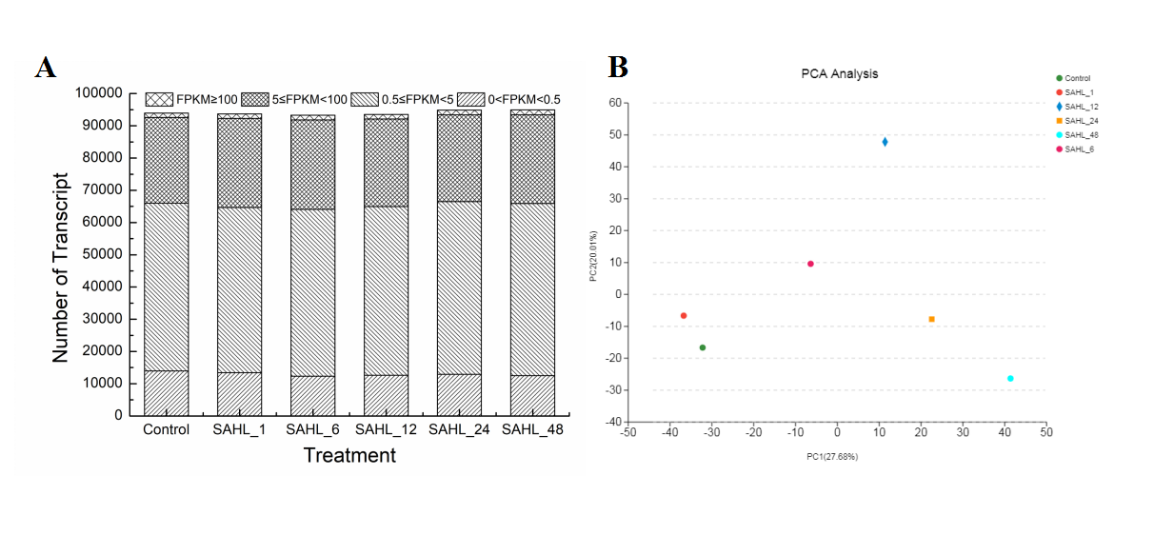


**Fig. S1.** Global gene expression profiling of *H. pluvialis* cells in different treatment stages. (A) Numbers of detected transcripts in each sample. (B) Principal component analysis of the RNA-Seq data.


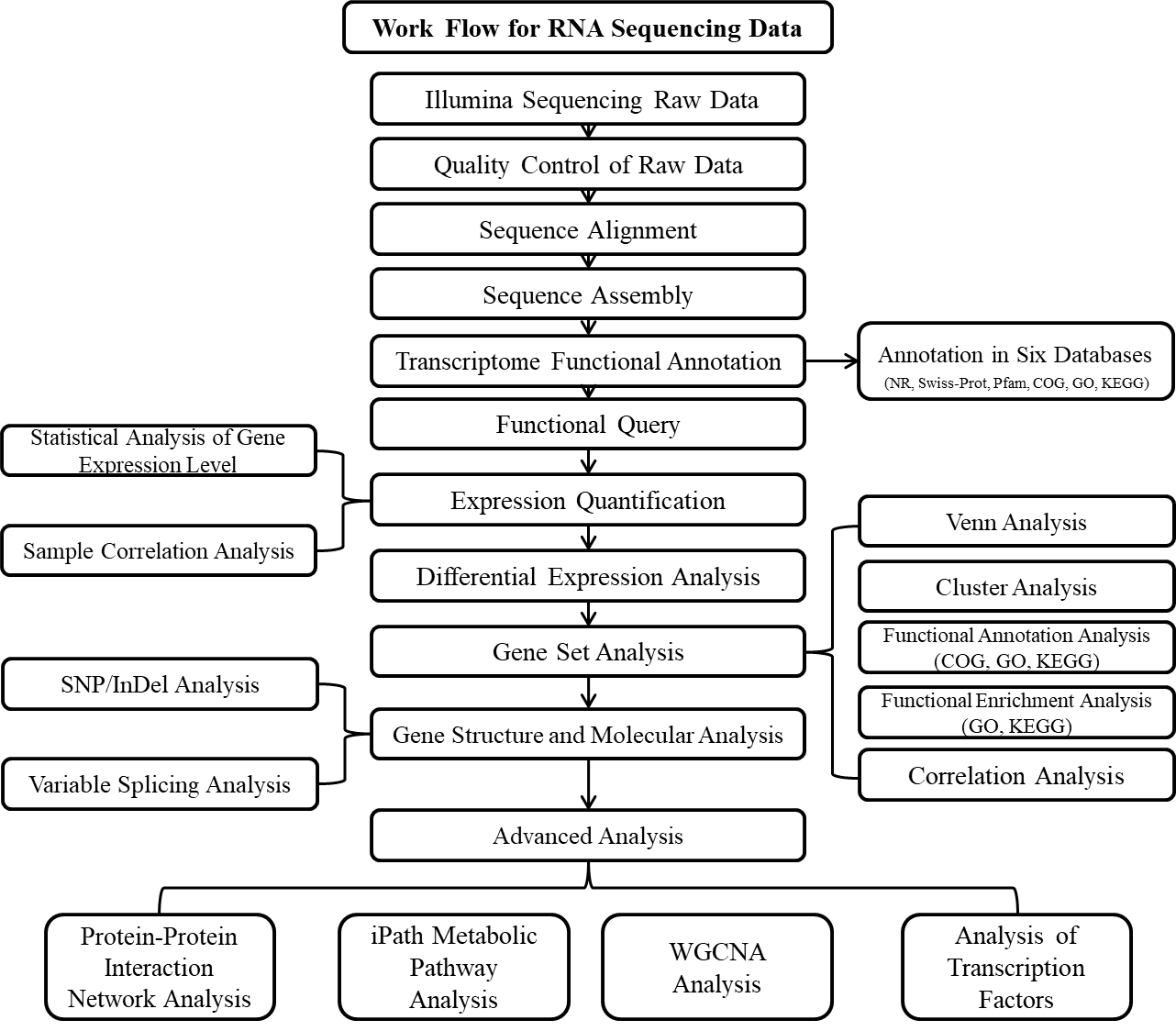


**Fig. S2.** Work flow of the methodology on transcriptome data analysis.

**Table S1** Changes in significantly enriched functional KEGG Pathways in *H. pluvialis* indentified by KEGG pathway classification during treatment stage transitions.

| Stage Transition | Change pattern | Description | Cluster Ratio % | P-value | P-adjust |
| --- | --- | --- | --- | --- | --- |
| SAHL_1 vs Control | up-regulated | Glyoxylate and dicarboxylate metabolism | 5.44 | 1.79E-12 | 1.79E-10 |
|  |  | ABC transporters | 5.07 | 6.44E-11 | 3.22E-09 |
|  |  | Nitrogen metabolism | 2.81 | 4.85E-10 | 1.62E-08 |
|  |  | Carotenoid biosynthesis | 2.63 | 3.03E-08 | 7.57E-07 |
|  |  | Alanine, aspartate and glutamate metabolism | 3 | 4.35E-05 | 8.69 E-04 |
|  |  | Biosynthesis of unsaturated fatty acids | 1.69 | 6.05E-05 | 1.01 E-03 |
|  |  | Starch and sucrose metabolism | 4.32 | 7.75E-05 | 1.11 E-03 |
|  |  | Pyruvate metabolism | 4.32 | 9.42E-05 | 1.18 E-03 |
|  |  | Biosynthesis of terpenoids and steroids | 0.56 | 1.11 E-04 | 1.23 E-03 |
|  |  | Butanoate metabolism | 1.88 | 2.35 E-04 | 2.35 E-03 |
|  |  | Arginine biosynthesis | 2.06 | 3.93 E-04 | 3.57 E-03 |
|  |  | Tyrosine metabolism | 1.69 | 6.04 E-04 | 4.65 E-03 |
|  |  | Peroxisome | 3.38 | 5.78 E-04 | 4.82 E-03 |
|  |  | Caffeine metabolism | 0.75 | 8.97 E-04 | 6.40 E-03 |
|  |  | Ubiquinone and other terpenoid-quinone biosynthesis | 1.5 | 1.06 E-03 | 7.06 E-03 |
|  |  | Arginine and proline metabolism | 2.06 | 2.87 E-03 | 0.02 |
|  |  | Glycolysis / Gluconeogenesis | 3.19 | 3.18 E-03 | 0.02 |
|  |  | Citrate cycle (TCA cycle) | 2.06 | 4.01 E-03 | 0.02 |
|  |  | Oxidative phosphorylation | 3.75 | 8.04 E-03 | 0.04 |
|  |  | Fatty acid degradation | 1.5 | 8.03 E-03 | 0.04 |
|  |  | Porphyrin and chlorophyll metabolism | 2.44 | 0.01 | 0.05 |
|  |  | Propanoate metabolism | 1.88 | 0.01 | 0.05 |
|  |  | One carbon pool by folate | 1.13 | 0.01 | 0.06 |
|  |  | Betalain biosynthesis | 0.38 | 0.04 | 0.14 |
|  |  | Fatty acid biosynthesis | 1.5 | 0.03 | 0.15 |
| SAHL_6 vs SAHL_1 | up-regulated | Tropane, piperidine and pyridine alkaloid biosynthesis | 2.06 | 2.71E-06 | 2.38E-04 |
|  |  | Tyrosine metabolism | 2.65 | 1.98E-05 | 8.72E-04 |
|  |  | Isoquinoline alkaloid biosynthesis | 1.77 | 4.36E-05 | 1.28 E-03 |
|  |  | Phenylalanine metabolism | 2.06 | 1.26E-04 | 2.21E-03 |
|  |  | Photosynthesis | 3.83 | 1.06E-04 | 2.32E-03 |
|  |  | Fatty acid elongation | 1.77 | 2.49E-04 | 3.66E-03 |
|  |  | Carbon fixation in photosynthetic organisms | 2.95 | 6.46E-03 | 0.08 |
|  |  | beta-Alanine metabolism | 1.47 | 0.02 | 0.16 |
|  |  | Cutin, suberine and wax biosynthesis | 0.59 | 0.02 | 0.17 |
|  |  | AGE-RAGE signaling pathway in diabetic complications | 1.47 | 0.03 | 0.24 |
|  |  | Vitamin B6 metabolism | 0.88 | 0.03 | 0.25 |
|  | down-regulated | Carotenoid biosynthesis | 2.94 | 4.49E-06 | 3.95 E-04 |
|  |  | ABC transporters | 3.92 | 1.92 E-04 | 8.44 E-03 |
|  |  | Caffeine metabolism | 0.98 | 2.08 E-03 | 0.05 |
|  |  | Arginine and proline metabolism | 2.61 | 2.64 E-03 | 0.05 |
|  |  | Biosynthesis of terpenoids and steroids | 0.65 | 1.80 E-03 | 0.05 |
|  |  | Purine metabolism | 6.54 | 0.01 | 0.18 |
|  |  | Phagosome | 2.61 | 0.02 | 0.23 |
|  |  | Other types of O-glycan biosynthesis | 1.63 | 0.03 | 0.32 |
|  |  | Glycolysis / Gluconeogenesis | 2.94 | 0.04 | 0.43 |
| SAHL_12 vs SAHL_6 | up-regulated | Glyoxylate and dicarboxylate metabolism | 6.71 | 5.64E-11 | 4.85E-09 |
|  |  | Pyruvate metabolism | 7.35 | 1.11E-08 | 4.78E-07 |
|  |  | Glycolysis / Gluconeogenesis | 5.11 | 2.38E-05 | 6.82E-04 |
|  |  | Alanine, aspartate and glutamate metabolism | 3.83 | 4.12E-05 | 8.85E-04 |
|  |  | Carbon fixation in photosynthetic organisms | 4.15 | 7.91E-05 | 1.36E-03 |
|  |  | Citrate cycle (TCA cycle) | 3.19 | 2.31E-04 | 3.32E-03 |
|  |  | Purine metabolism | 7.35 | 1.87E-03 | 0.02 |
|  |  | beta-Alanine metabolism | 1.92 | 2.42E-03 | 0.03 |
|  |  | Propanoate metabolism | 2.56 | 3.78E-03 | 0.04 |
|  |  | Pyrimidine metabolism | 4.15 | 0.01 | 0.12 |
|  |  | Thiamine metabolism | 1.28 | 0.02 | 0.13 |
|  |  | Biosynthesis of unsaturated fatty acids | 1.28 | 0.02 | 0.14 |
|  |  | Cysteine and methionine metabolism | 2.56 | 0.02 | 0.15 |
|  |  | Arginine and proline metabolism | 1.92 | 0.03 | 0.21 |
|  |  | Fatty acid elongation | 0.96 | 0.05 | 0.28 |
|  | down-regulated | ABC transporters | 6.54 | 2.89E-10 | 2.63E-08 |
|  |  | Phenylalanine metabolism | 3.27 | 5.86E-08 | 2.67E-06 |
|  |  | Tropane, piperidine and pyridine alkaloid biosynthesis | 2.29 | 1.37E-06 | 4.16E-05 |
|  |  | Isoquinoline alkaloid biosynthesis | 1.96 | 2.45E-05 | 5.58E-04 |
|  |  | beta-Alanine metabolism | 2.29 | 3.69E-04 | 6.71E-03 |
|  |  | Starch and sucrose metabolism | 4.58 | 1.13E-03 | 0.02 |
|  |  | Biosynthesis of terpenoids and steroids | 0.65 | 1.80E-03 | 0.02 |
|  |  | Carotenoid biosynthesis | 1.96 | 1.64E-03 | 0.02 |
|  |  | Arachidonic acid metabolism | 1.96 | 2.16E-03 | 0.02 |
|  |  | Tyrosine metabolism | 1.96 | 2.46E-03 | 0.02 |
|  |  | Photosynthesis | 2.94 | 7.12E-03 | 0.06 |
|  |  | Linoleic acid metabolism | 0.65 | 0.01 | 0.08 |
|  |  | Thermogenesis | 4.9 | 0.01 | 0.08 |
|  |  | Tryptophan metabolism | 1.31 | 0.02 | 0.10 |
|  |  | Circadian rhythm - plant | 0.98 | 0.02 | 0.10 |
|  |  | Phenylpropanoid biosynthesis | 0.65 | 0.02 | 0.12 |
|  |  | Selenocompound metabolism | 1.31 | 0.02 | 0.13 |
|  |  | Folate biosynthesis | 1.63 | 0.03 | 0.15 |
| SAHL_24 vs SAHL_12 | up-regulated | ABC transporters | 5.73 | 4.39E-10 | 3.87E-08 |
|  |  | Tropane, piperidine and pyridine alkaloid biosynthesis | 2.6 | 1.39E-09 | 6.12E-08 |
|  |  | Phenylalanine metabolism | 2.86 | 4.55E-08 | 1.34E-06 |
|  |  | Biosynthesis of terpenoids and steroids | 1.04 | 2.3E-07 | 5.07E-06 |
|  |  | Isoquinoline alkaloid biosynthesis | 2.08 | 5.6E-07 | 9.86E-06 |
|  |  | Carotenoid biosynthesis | 2.6 | 3.75E-06 | 5.51E-05 |
|  |  | Tyrosine metabolism | 2.6 | 7.84E-06 | 9.85E-05 |
|  |  | Selenocompound metabolism | 2.08 | 6.86E-05 | 7.54E-04 |
|  |  | beta-Alanine metabolism | 1.82 | 1.40E-03 | 0.01 |
|  |  | Starch and sucrose metabolism | 4.17 | 1.37E-03 | 0.01 |
|  |  | Plant hormone signal transduction | 1.82 | 3.05E-03 | 0.02 |
|  |  | Photosynthesis - antenna proteins | 1.82 | 2.86E-03 | 0.02 |
|  |  | Thermogenesis | 4.69 | 9.33E-03 | 0.06 |
|  |  | Photosynthesis | 2.34 | 0.03 | 0.16 |
|  |  | Oxidative phosphorylation | 3.65 | 0.03 | 0.16 |
|  |  | Glycine, serine and threonine metabolism | 2.34 | 0.03 | 0.17 |
|  |  | Porphyrin and chlorophyll metabolism | 2.34 | 0.04 | 0.20 |
|  |  | Phenylalanine, tyrosine and tryptophan biosynthesis | 1.3 | 0.04 | 0.21 |
|  | down-regulated | Ribosome biogenesis in eukaryotes | 8.1 | 2.19E-12 | 1.93E-10 |
|  |  | Nitrogen metabolism | 3.43 | 1.8E-08 | 7.91E-07 |
|  |  | Alanine, aspartate and glutamate metabolism | 4.98 | 6.2E-08 | 1.82E-06 |
|  |  | Glyoxylate and dicarboxylate metabolism | 5.3 | 1.35E-07 | 2.97E-06 |
|  |  | Pyruvate metabolism | 4.05 | 5.30E-03 | 0.08 |
|  |  | Citrate cycle (TCA cycle) | 2.49 | 4.59E-03 | 0.08 |
|  |  | Purine metabolism | 6.54 | 0.01 | 0.13 |
|  |  | beta-Alanine metabolism | 1.56 | 0.01 | 0.14 |
|  |  | Carbon fixation in photosynthetic organisms | 2.49 | 0.03 | 0.34 |
| SAHL_48 vs SAHL_24 | up-regulated | Fatty acid elongation | 2.25 | 6.78E-05 | 5.49E-03 |
|  |  | Fructose and mannose metabolism | 3 | 2.09E-04 | 8.46E-03 |
|  |  | Cutin, suberine and wax biosynthesis | 1.12 | 3.92E-04 | 0.01 |
|  |  | Amino sugar and nucleotide sugar metabolism | 3 | 3.65E-03 | 0.07 |
|  |  | Endocytosis | 3.75 | 6.88E-03 | 0.11 |
|  |  | Photosynthesis | 3 | 9.84E-03 | 0.13 |
|  |  | Carbon fixation in photosynthetic organisms | 2.62 | 0.04 | 0.43 |
|  |  | AGE-RAGE signaling pathway in diabetic complications | 1.5 | 0.05 | 0.48 |
|  | down-regulated | ABC transporters | 4.24 | 8.07E-07 | 8.07E-05 |
|  |  | Photosynthesis | 3.13 | 4.74E-04 | 0.02 |
|  |  | Fatty acid elongation | 1.34 | 1.08E-03 | 0.04 |
|  |  | Cutin, suberine and wax biosynthesis | 0.67 | 1.76E-03 | 0.04 |
|  |  | Amino sugar and nucleotide sugar metabolism | 2.46 | 3.28E-03 | 0.07 |
|  |  | Fructose and mannose metabolism | 1.79 | 5.63E-03 | 0.09 |
|  |  | Carbon fixation in photosynthetic organisms | 2.46 | 0.02 | 0.23 |
|  |  | AGE-RAGE signaling pathway in diabetic complications | 1.34 | 0.03 | 0.34 |
|  |  | Oxidative phosphorylation | 3.35 | 0.05 | 0.53 |

**Table S2** The 10 significantly expressed profiles and their significantly enriched functional pathways.

| Profile | Pathway Description | Cluster frequency (%) | P-value |
| --- | --- | --- | --- |
| profile 0 | Phagosome | 5.46 | 7.35E-09 |
|  | Relaxin signaling pathway | 3.74 | 2.92E-07 |
|  | AGE-RAGE signaling pathway in diabetic complications | 2.59 | 7.01E-05 |
|  | MAPK signaling pathway - plant | 2.59 | 3.62E-03 |
|  | Human cytomegalovirus infection | 2.01 | 7.15E-03 |
| profile 13 | Fatty acid elongation | 1.97 | 7.47E-03 |
|  | Cellular senescence | 3.95 | 6.42E-03 |
|  | C-type lectin receptor signaling pathway | 1.97 | 5.05E-03 |
| profile 18 | Glycerolipid metabolism | 3.59 | 7.26E-04 |
|  | ABC transporters | 4.79 | 6.26E-04 |
|  | Circadian rhythm - plant | 1.8 | 2.89E-03 |
| profile 30 | Isoquinoline alkaloid biosynthesis | 3.42 | 7.61E-05 |
|  | beta-Alanine metabolism | 4.27 | 1.57E-04 |
|  | Tropane, piperidine and pyridine alkaloid biosynthesis | 3.42 | 6.75E-05 |
|  | Phenylalanine metabolism | 3.42 | 6.15E-04 |
|  | Tyrosine metabolism | 3.42 | 1.87E-03 |
| profile 31 | Ribosome biogenesis in eukaryotes | 6.44 | 3.89E-09 |
|  | Citrate cycle (TCA cycle) | 3.36 | 3.33E-05 |
|  | Purine metabolism | 7.84 | 2.22E-04 |
|  | Alanine, aspartate and glutamate metabolism | 3.36 | 1.44E-04 |
|  | Glyoxylate and dicarboxylate metabolism | 3.64 | 2.05E-04 |
|  | Phenylalanine, tyrosine and tryptophan biosynthesis | 1.96 | 2.03E-03 |
|  | Cysteine and methionine metabolism | 3.08 | 1.96E-03 |
|  | Carbon fixation in photosynthetic organisms | 3.08 | 3.15E-03 |
|  | Pyruvate metabolism | 3.92 | 5.14E-03 |
|  | Isoquinoline alkaloid biosynthesis | 1.12 | 4.91E-03 |
|  | Lysine biosynthesis | 1.4 | 4.84E-03 |
|  | Tropane, piperidine and pyridine alkaloid biosynthesis | 1.12 | 4.40E-03 |
| profile 40 | ABC transporters | 7.79 | 1.8E-07 |
|  | Parathyroid hormone synthesis, secretion and action | 3.25 | 6.31E-03 |
| profile 44 | Glyoxylate and dicarboxylate metabolism | 7.43 | 9.02E-09 |
|  | Citrate cycle (TCA cycle) | 3.96 | 2.38E-04 |
|  | Indole alkaloid biosynthesis | 0.99 | 1.94E-03 |
|  | Tryptophan metabolism | 1.98 | 3.74E-03 |
|  | Photosynthesis - antenna proteins | 2.48 | 3.22E-03 |
|  | Propanoate metabolism | 2.97 | 5.80E-03 |
|  | Cysteine and methionine metabolism | 3.47 | 6.90E-03 |
|  | Betalain biosynthesis | 0.99 | 5.64E-03 |
| profile 48 | Nitrogen metabolism | 4 | 1.08E-07 |
|  | Alanine, aspartate and glutamate metabolism | 5.33 | 1.47E-06 |
|  | Fatty acid degradation | 4 | 3.44E-06 |
|  | Tyrosine metabolism | 3.56 | 7.26E-06 |
|  | Glycolysis / Gluconeogenesis | 5.33 | 1.68E-04 |
|  | alpha-Linolenic acid metabolism | 1.78 | 1.87E-03 |
|  | Indole alkaloid biosynthesis | 0.89 | 2.40E-03 |
|  | Betalain biosynthesis | 0.89 | 6.95E-03 |
|  | Isoquinoline alkaloid biosynthesis | 1.33 | 9.42E-03 |
| profile 49 | DNA replication | 5.88 | 2.62E-12 |
|  | Fatty acid degradation | 2.45 | 6.85E-05 |
|  | Fatty acid elongation | 1.47 | 6.67E-04 |
|  | Cutin, suberine and wax biosynthesis | 0.74 | 1.35E-03 |
|  | Riboflavin metabolism | 1.23 | 7.18E-03 |

**Table S3** Transcription factor families identified in *H. pluvialis* in this study.

| TF family | Gene number | Transcript number |
| --- | --- | --- |
| C3H | 51 | 113 |
| MYB | 44 | 80 |
| Nin-like | 44 | 69 |
| MYB_related | 35 | 57 |
| ERF | 30 | 59 |
| bZIP | 25 | 48 |
| GATA | 22 | 42 |
| SBP | 21 | 37 |
| AP2 | 20 | 35 |
| bHLH | 13 | 27 |
| CPP | 13 | 23 |
| C2H2 | 10 | 15 |
| B3 | 8 | 14 |
| HB-other | 6 | 8 |
| WRKY | 5 | 9 |
| HSF | 5 | 7 |
| E2F/DP | 5 | 6 |
| M_type | 5 | 5 |
| NF-X1 | 3 | 14 |
| Whirly | 3 | 9 |
| LSD | 3 | 6 |
| CO-like | 3 | 5 |
| DBB | 3 | 5 |
| Dof | 3 | 4 |
| YABBY | 3 | 3 |

**Table S4** Genes involved in astaxanthin and fatty acids biosynthesis pathways and their expression level of FPKM in *H. pluvialis* in this study.

| Gene description | Gene_id | Control | SAHL_1 | SAHL_6 | SAHL_12 | SAHL_24 | SAHL_48 |
| --- | --- | --- | --- | --- | --- | --- | --- |
| isopentenyl pyrophosphate isomerase (IPI) | MSTRG.64143 | 16.9 | 20.79 | 20.5 | 21.62 | 21.28 | 19.76 |
| chloroplast geranylgeranyl diphosphate synthase (GGPS) | Ch_GLEAN_10010046 | 4.29 | 8.69 | 5.09 | 8.37 | 7.01 | 4.18 |
|  | MSTRG.11757 | 32.78 | 34.16 | 51.19 | 56.92 | 55.54 | 24.34 |
|  | MSTRG.61586 | 33.98 | 39.09 | 38.41 | 33.46 | 48.02 | 35.35 |
|  | MSTRG.66739 | 28.92 | 29.7 | 26.85 | 28.66 | 30.79 | 28.33 |
| phytoene synthase (PSY) | Ch_GLEAN_10005596 | 7.82 | 12.6 | 8.13 | 8.45 | 10.06 | 5.69 |
|  | Ch_GLEAN_10007856 | 53.99 | 57.1 | 67.06 | 60.48 | 65.25 | 60.93 |
|  | Ch_GLEAN_10007976 | 14.97 | 14.27 | 11.81 | 15.08 | 16.24 | 15.35 |
|  | Ch_GLEAN_10009230 | 6.39 | 7.09 | 4.95 | 5.16 | 6.68 | 3.21 |
|  | Ch_GLEAN_10011828 | 43.59 | 36.17 | 43.81 | 47.88 | 52.8 | 49.72 |
|  | Ch_GLEAN_10011841 | 29.28 | 26.75 | 30.6 | 29.59 | 34.46 | 29.19 |
|  | Ch_GLEAN_10011843 | 0 | 0 | 0.05 | 0.82 | 0 | 0.77 |
|  | MSTRG.133 | 0.12 | 0 | 0.07 | 0 | 0.25 | 0.26 |
|  | MSTRG.134 | 4.31 | 8.27 | 0 | 2.95 | 15.59 | 0 |
|  | MSTRG.54158 | 8.87 | 17.28 | 8.9 | 6.11 | 17.65 | 14.86 |
|  | MSTRG.61209 | 3.78 | 14.89 | 12.36 | 5.74 | 18.75 | 10.62 |
|  | MSTRG.61210 | 6.27 | 21.62 | 10.72 | 2.4 | 17.22 | 10.25 |
|  | MSTRG.61211 | 5.17 | 11.55 | 5.2 | 3.26 | 8.93 | 10.2 |
| phytoene desaturase (PDS) | Ch_GLEAN_10001767 | 12.3 | 13.17 | 12.23 | 11.4 | 11.96 | 10.06 |
|  | Ch_GLEAN_10003923 | 28.77 | 26.38 | 28.29 | 25.71 | 22.11 | 30.37 |
|  | Ch_GLEAN_10011505 | 77.76 | 146.31 | 92.25 | 81.79 | 93.96 | 81.61 |
|  | Ch_GLEAN_10012094 | 15.41 | 18.38 | 16.59 | 21.58 | 21.82 | 24.19 |
|  | MSTRG.2769 | 0.77 | 2.89 | 1.81 | 0.44 | 1.42 | 0.21 |
|  | MSTRG.8263 | 53.63 | 194.76 | 91.29 | 56.08 | 103.13 | 84.94 |
|  | MSTRG.8264 | 0 | 0 | 0 | 0 | 0 | 0.06 |
| chloroplast zeta-carotene desaturase (ZDS) | Ch_GLEAN_10009908 | 10.68 | 33.1 | 16.08 | 10.27 | 17.29 | 12.97 |
|  | Ch_GLEAN_10010991 | 16.48 | 50.98 | 22.23 | 15.76 | 25.43 | 17.92 |
|  | MSTRG.19044 | 20.26 | 61.47 | 25.01 | 16.11 | 27.91 | 14.47 |
|  | MSTRG.20587 | 0 | 0 | 1.07 | 0 | 0 | 1.76 |
|  | MSTRG.28397 | 0 | 0 | 3.25 | 0 | 0 | 0 |
|  | MSTRG.38225 | 6.63 | 20.95 | 9.9 | 5.54 | 10.52 | 7.48 |
|  | MSTRG.38226 | 18.25 | 64.15 | 25.6 | 17.24 | 26.44 | 20.27 |
| lycopene beta cyclase (LCY) | Ch_GLEAN_10007045 | 18.21 | 42.14 | 29.67 | 16.09 | 33.94 | 31.73 |
|  | Ch_GLEAN_10007046 | 0 | 0 | 0 | 0.77 | 0.13 | 0.59 |
|  | Ch_GLEAN_10010036 | 13.86 | 34.24 | 27.58 | 11.37 | 22.41 | 22.25 |
|  | Ch_GLEAN_10010071 | 86.05 | 120.53 | 121.22 | 97.24 | 118.98 | 135.87 |
| beta-carotene hydroxylase (CRTO) | Ch_GLEAN_10006000 | 8.04 | 8.18 | 7.18 | 7.79 | 5.18 | 7.97 |
|  | Ch_GLEAN_10011708 | 4.58 | 3.72 | 1.95 | 2.3 | 4.6 | 4.7 |
|  | MSTRG.66299 | 1.38 | 1.13 | 0.45 | 1 | 2.16 | 3.92 |
| beta-carotene ketolase (BKT) | Ch_GLEAN_10009207 | 27.97 | 23.92 | 35.26 | 42.06 | 37.5 | 43.72 |
|  | Ch_GLEAN_10009310 | 18.22 | 22.77 | 20.87 | 23.85 | 24.09 | 24.2 |
|  | MSTRG.24214 | 0 | 0 | 0 | 0.36 | 0 | 0 |
|  | MSTRG.24215 | 0.28 | 1.78 | 0.57 | 1.26 | 1 | 1.42 |
|  | MSTRG.37469 | 1.89 | 10.97 | 7.74 | 4.47 | 9.73 | 7.48 |
|  | MSTRG.37470 | 1.61 | 2.85 | 4.12 | 2.42 | 1.68 | 1.95 |
|  | MSTRG.49769 | 1.17 | 2.94 | 2.49 | 1.28 | 2.74 | 2.56 |
|  | MSTRG.49770 | 1.33 | 5.5 | 3.01 | 0.83 | 3.04 | 3.06 |
|  | MSTRG.50648 | 3.01 | 4.1 | 3.24 | 2.58 | 3.13 | 4.97 |
| carotenoid hydroxylase (CRTR-B) | Ch_GLEAN_10000440 | 0 | 0 | 0 | 0 | 0 | 0 |
|  | MSTRG.29170 | 3.45 | 2.25 | 3.26 | 2.5 | 3.39 | 2.43 |
| biotin carboxylase (BC) | Ch_GLEAN_10000659 | 45.9 | 91.29 | 71.27 | 45.17 | 51.13 | 39.28 |
|  | Ch_GLEAN_10001836 | 2.87 | 6.64 | 8.48 | 7.07 | 6.39 | 8.15 |
|  | Ch_GLEAN_10004011 | 18.69 | 11.39 | 21.14 | 8.92 | 10.04 | 10.03 |
|  | Ch_GLEAN_10004721 | 84.8 | 94.24 | 86.8 | 81.01 | 81.02 | 67 |
|  | Ch_GLEAN_10006331 | 8.19 | 3.9 | 4.82 | 5.12 | 5.33 | 5.69 |
|  | Ch_GLEAN_10006501 | 83.2 | 85.72 | 75.98 | 69.41 | 66.29 | 59.38 |
|  | Ch_GLEAN_10010198 | 123.49 | 82.87 | 129.89 | 80.24 | 80.02 | 72.84 |
|  | MSTRG.10057 | 0 | 0 | 0 | 0 | 0 | 0 |
|  | MSTRG.11586 | 35.04 | 39.6 | 26.84 | 36.33 | 35.04 | 34.85 |
|  | MSTRG.1708 | 0 | 0.91 | 0 | 1.58 | 0.24 | 1.21 |
|  | MSTRG.17869 | 1.03 | 0.26 | 0.94 | 0 | 1.08 | 1.9 |
|  | MSTRG.17870 | 54.22 | 72.56 | 61.65 | 62.49 | 57.71 | 49.6 |
|  | MSTRG.23960 | 8.41 | 9.63 | 10.96 | 6.92 | 6.76 | 10.12 |
|  | MSTRG.25433 | 23.86 | 26.47 | 22.58 | 22.55 | 24.24 | 20.29 |
|  | MSTRG.35199 | 10.45 | 9.8 | 9.11 | 9.45 | 11.23 | 9.69 |
|  | MSTRG.41884 | 0 | 14.97 | 0 | 5.82 | 6.61 | 0 |
|  | MSTRG.45663 | 51.91 | 51.89 | 36.96 | 45.98 | 47.69 | 42.07 |
|  | MSTRG.45664 | 22.46 | 26.95 | 19.5 | 27.02 | 21.45 | 14.08 |
|  | MSTRG.50807 | 54.37 | 63.22 | 42.87 | 53.3 | 53.2 | 45 |
|  | MSTRG.54350 | 56.95 | 53.83 | 46.97 | 59.11 | 54.99 | 45.21 |
|  | MSTRG.54351 | 24.7 | 54.93 | 58.64 | 28.09 | 19.96 | 40.1 |
|  | MSTRG.5955 | 81.34 | 89.04 | 95.58 | 97.19 | 95.71 | 82.11 |
|  | MSTRG.59823 | 7.46 | 4.02 | 7.79 | 4.46 | 4.73 | 3.57 |
|  | MSTRG.59824 | 2.96 | 6.74 | 0 | 0 | 2.2 | 1.47 |
|  | MSTRG.7199 | 4.41 | 4.08 | 5.28 | 4.24 | 3.18 | 6.87 |
|  | MSTRG.7947 | 2.49 | 5.19 | 5.06 | 4.94 | 3.11 | 3.82 |
|  | MSTRG.7949 | 0 | 0 | 4.06 | 0 | 0 | 0 |
|  | MSTRG.7950 | 6.22 | 8.71 | 7.5 | 6.3 | 6.39 | 6.87 |
| acyl carrier protein (ACP) | Ch_GLEAN_10000181 | 0 | 0 | 0 | 0 | 0 | 0 |
|  | Ch_GLEAN_10000643 | 0 | 0 | 0 | 0 | 0 | 0 |
|  | Ch_GLEAN_10001844 | 15.79 | 20.11 | 22.39 | 17.92 | 23.18 | 28.05 |
|  | Ch_GLEAN_10002269 | 0 | 0 | 0 | 0 | 0 | 0 |
|  | Ch_GLEAN_10002495 | 12.92 | 10.96 | 12.61 | 11.57 | 13.87 | 11.55 |
|  | Ch_GLEAN_10002614 | 13.06 | 7.42 | 11.19 | 10.93 | 13 | 14.92 |
|  | Ch_GLEAN_10002742 | 21.18 | 13.51 | 17.41 | 12.44 | 18.21 | 18.17 |
|  | Ch_GLEAN_10002771 | 10.83 | 59.15 | 40.67 | 18.08 | 35.73 | 29.97 |
|  | Ch_GLEAN_10002956 | 18.67 | 18.85 | 21.33 | 19.76 | 22.64 | 23.24 |
|  | Ch_GLEAN_10003108 | 0 | 0.83 | 2.5 | 0.14 | 0 | 1.78 |
|  | Ch_GLEAN_10003626 | 44.61 | 36.02 | 37.12 | 33.29 | 36.59 | 40.91 |
|  | Ch_GLEAN_10003639 | 9.97 | 15.03 | 12.85 | 12.29 | 15.2 | 17.82 |
|  | Ch_GLEAN_10003831 | 276.31 | 272.76 | 268.91 | 267.09 | 256.23 | 303.97 |
|  | Ch_GLEAN_10003941 | 16.96 | 46.52 | 29.85 | 28.8 | 34.13 | 27.95 |
|  | Ch_GLEAN_10004007 | 10.18 | 53.76 | 34.99 | 14.67 | 33.21 | 26.57 |
|  | Ch_GLEAN_10004079 | 25.93 | 17.93 | 19.96 | 18.18 | 18.42 | 14.16 |
|  | Ch_GLEAN_10004636 | 52.78 | 34.82 | 49.34 | 64.42 | 42.87 | 41.45 |
|  | Ch_GLEAN_10005332 | 1.25 | 7.43 | 4.42 | 4.33 | 6.96 | 6.27 |
|  | Ch_GLEAN_10005409 | 28.4 | 24.93 | 24.79 | 33.73 | 33.51 | 28.26 |
|  | Ch_GLEAN_10005475 | 19.76 | 21.81 | 24.57 | 31.88 | 32.11 | 30.9 |
|  | Ch_GLEAN_10005522 | 45.58 | 73.99 | 66.78 | 56.57 | 66.35 | 55.3 |
|  | Ch_GLEAN_10005843 | 5.57 | 9.38 | 17.8 | 6.58 | 20.79 | 22.02 |
|  | Ch_GLEAN_10005982 | 30.88 | 27.18 | 34.19 | 33.87 | 32.87 | 33.09 |
|  | Ch_GLEAN_10006059 | 1.16 | 0 | 2.66 | 6.06 | 4.05 | 2.26 |
|  | Ch_GLEAN_10006151 | 10.92 | 9.74 | 9.95 | 9.69 | 10.47 | 8.43 |
|  | Ch_GLEAN_10006327 | 2.17 | 1.78 | 2.73 | 2.92 | 2.16 | 4.11 |
|  | Ch_GLEAN_10006380 | 37.88 | 36.41 | 43.88 | 31.14 | 43.53 | 38.51 |
|  | Ch_GLEAN_10006911 | 16.56 | 14.55 | 13.93 | 13.99 | 17.01 | 18.79 |
|  | Ch_GLEAN_10006981 | 11.74 | 11.05 | 11.63 | 11.42 | 12.29 | 6.84 |
|  | Ch_GLEAN_10007431 | 48.68 | 68.94 | 70.32 | 86.68 | 75.41 | 72.07 |
|  | Ch_GLEAN_10007838 | 8.71 | 3.93 | 8.23 | 7.74 | 7.5 | 7.44 |
|  | Ch_GLEAN_10008025 | 3.97 | 3.16 | 3.42 | 4.34 | 4.41 | 3.88 |
|  | Ch_GLEAN_10008565 | 48.8 | 20.11 | 15.79 | 22.25 | 15.28 | 22.17 |
|  | Ch_GLEAN_10008884 | 29.03 | 32.42 | 24.95 | 31.12 | 32.08 | 22.75 |
|  | Ch_GLEAN_10009155 | 3.7 | 9.06 | 14.41 | 19.31 | 21.13 | 16.61 |
|  | Ch_GLEAN_10009434 | 13.13 | 8.19 | 8.06 | 8.95 | 16.6 | 10.83 |
|  | Ch_GLEAN_10010082 | 8.23 | 17.7 | 28.24 | 15.18 | 28.35 | 53.82 |
|  | Ch_GLEAN_10010090 | 26.83 | 23.56 | 18.15 | 24.85 | 24.44 | 33.17 |
|  | Ch_GLEAN_10010364 | 28.11 | 32.96 | 37.73 | 40.21 | 35.77 | 34.2 |
|  | Ch_GLEAN_10010568 | 18.05 | 16.22 | 17.41 | 17.42 | 19.19 | 19.21 |
|  | Ch_GLEAN_10010664 | 17.52 | 14.41 | 22.11 | 19.15 | 20.88 | 21.6 |
|  | Ch_GLEAN_10010676 | 26.39 | 21.79 | 24.74 | 17.63 | 21.44 | 23.07 |
|  | Ch_GLEAN_10010782 | 8.86 | 9.48 | 11.91 | 7.91 | 8.6 | 9.87 |
|  | Ch_GLEAN_10010915 | 37.57 | 29.34 | 29.51 | 26 | 29.66 | 28.09 |
|  | Ch_GLEAN_10011051 | 33.02 | 25.6 | 26.84 | 31.14 | 29.98 | 27.76 |
|  | Ch_GLEAN_10011171 | 6.4 | 4.88 | 3.93 | 4.22 | 4.32 | 3.35 |
|  | Ch_GLEAN_10011866 | 11.56 | 11.21 | 13.43 | 10.26 | 10.11 | 10.27 |
|  | Ch_GLEAN_10012081 | 21.47 | 103.03 | 66.04 | 37.21 | 62.08 | 49.85 |
|  | Ch_GLEAN_10012091 | 39.58 | 37.04 | 31.36 | 42.19 | 41.74 | 36.29 |
|  | Ch_GLEAN_10012146 | 42.08 | 37.7 | 39.29 | 44.32 | 42.1 | 40.66 |
|  | Ch_GLEAN_10012312 | 2.08 | 17.65 | 16.7 | 6.36 | 18.43 | 16.18 |
|  | Ch_GLEAN_10012421 | 18.1 | 55.94 | 30.21 | 20.02 | 28.83 | 19.99 |
|  | MSTRG.10492 | 7.26 | 8.08 | 9.28 | 6.11 | 11.51 | 9.51 |
|  | MSTRG.10767 | 1.3 | 1.52 | 2.67 | 1.27 | 1.98 | 2.6 |
|  | MSTRG.11053 | 3.27 | 8.89 | 5.56 | 5.91 | 8.3 | 4.83 |
|  | MSTRG.12646 | 1.4 | 1.24 | 3.28 | 0.48 | 3.07 | 4.26 |
|  | MSTRG.13187 | 0 | 0.05 | 1.14 | 0 | 0.71 | 1.22 |
|  | MSTRG.13264 | 14.17 | 9.64 | 11.71 | 15.05 | 14.53 | 13.56 |
|  | MSTRG.13664 | 23.81 | 23.14 | 34.55 | 28.68 | 36.6 | 30.4 |
|  | MSTRG.14396 | 8.27 | 5.59 | 4.76 | 9.16 | 8.57 | 9.31 |
|  | MSTRG.14398 | 38.05 | 21.97 | 30.95 | 38.74 | 25.82 | 26.73 |
|  | MSTRG.14716 | 22.7 | 27.12 | 17.9 | 26.69 | 26.32 | 20.51 |
|  | MSTRG.16143 | 136.17 | 200.36 | 129.47 | 164.84 | 145.32 | 115.14 |
|  | MSTRG.17537 | 4.12 | 7.21 | 5.55 | 12.43 | 10.87 | 10.05 |
|  | MSTRG.17978 | 0 | 0.14 | 1.2 | 0 | 0.61 | 0 |
|  | MSTRG.18602 | 3.03 | 3.1 | 3.42 | 4.71 | 4.35 | 3.34 |
|  | MSTRG.18603 | 0 | 0 | 0 | 0 | 0 | 0 |
|  | MSTRG.18970 | 25.09 | 23.75 | 25 | 25.44 | 21.78 | 20.33 |
|  | MSTRG.20674 | 0.8 | 0.31 | 0.57 | 1.74 | 0 | 2.07 |
|  | MSTRG.2152 | 4.19 | 3.55 | 4.35 | 5.29 | 5.21 | 4.82 |
|  | MSTRG.21735 | 0.34 | 0.32 | 0.85 | 0.49 | 0.3 | 0.8 |
|  | MSTRG.21987 | 12.71 | 7.9 | 6.94 | 6.84 | 4.76 | 5.25 |
|  | MSTRG.23195 | 12.87 | 11.98 | 14.07 | 12.14 | 13.14 | 13.84 |
|  | MSTRG.24085 | 11.78 | 11.99 | 16.41 | 11.3 | 15.22 | 14.53 |
|  | MSTRG.24086 | 16.88 | 22.57 | 31.41 | 18.72 | 22.66 | 22.95 |
|  | MSTRG.24657 | 0.89 | 0.92 | 3.24 | 2.52 | 2.47 | 2.97 |
|  | MSTRG.25051 | 97.45 | 82.93 | 77.89 | 66.12 | 78.49 | 76.51 |
|  | MSTRG.25525 | 7.52 | 7.09 | 7.16 | 10.39 | 5.86 | 5.75 |
|  | MSTRG.25916 | 1.42 | 1.66 | 1.24 | 1.39 | 1.56 | 1.91 |
|  | MSTRG.26629 | 71.12 | 68.61 | 53.68 | 68.93 | 68.77 | 57.89 |
|  | MSTRG.26630 | 0.06 | 0.06 | 0.2 | 0.13 | 0.19 | 0.31 |
|  | MSTRG.29131 | 0.95 | 0.38 | 2.03 | 0.78 | 1.76 | 4.11 |
|  | MSTRG.29359 | 10.56 | 10.83 | 11.03 | 13.01 | 11.43 | 10.78 |
|  | MSTRG.29623 | 2.51 | 6.92 | 14.28 | 2.89 | 16.15 | 62.68 |
|  | MSTRG.3005 | 2.23 | 1.66 | 2.05 | 2.1 | 2.38 | 2 |
|  | MSTRG.3227 | 1.83 | 2.27 | 2.41 | 2.31 | 3.47 | 2.75 |
|  | MSTRG.32760 | 51.92 | 52.37 | 43.34 | 42.07 | 40.22 | 42.01 |
|  | MSTRG.33070 | 2.03 | 5.57 | 9.87 | 7.19 | 1.41 | 2.89 |
|  | MSTRG.33072 | 1.88 | 1.48 | 1.6 | 2.72 | 2.53 | 3.11 |
|  | MSTRG.33860 | 13.2 | 23.85 | 25.42 | 26.21 | 31.85 | 30.16 |
|  | MSTRG.34844 | 28.77 | 45.79 | 43.03 | 31.88 | 42.67 | 31.75 |
|  | MSTRG.3554 | 67.7 | 60.82 | 63.27 | 74.74 | 72.01 | 75.44 |
|  | MSTRG.3669 | 13.12 | 11.43 | 12.62 | 11.23 | 11.44 | 8.36 |
|  | MSTRG.39451 | 0.46 | 0.72 | 1.19 | 0.63 | 0.67 | 1.53 |
|  | MSTRG.41629 | 37.6 | 47.73 | 39.13 | 45.17 | 46.07 | 37.97 |
|  | MSTRG.41714 | 0 | 0 | 0 | 0 | 0 | 0 |
|  | MSTRG.41715 | 9.92 | 8 | 9.93 | 17.79 | 13.96 | 10.42 |
|  | MSTRG.41716 | 9.55 | 8.38 | 10.82 | 16.09 | 12.91 | 13.42 |
|  | MSTRG.4221 | 8.6 | 8.99 | 10.04 | 9.25 | 9.8 | 9.97 |
|  | MSTRG.42581 | 14.18 | 4.9 | 10 | 12.07 | 8.66 | 15.23 |
|  | MSTRG.42584 | 0 | 0 | 0 | 0 | 0 | 0 |
|  | MSTRG.43421 | 22.84 | 25.39 | 25 | 25.68 | 23.44 | 21.12 |
|  | MSTRG.44609 | 64.42 | 66.32 | 60.57 | 89.29 | 82.77 | 58.15 |
|  | MSTRG.45359 | 162.75 | 152.79 | 155.91 | 172.65 | 172.9 | 190.91 |
|  | MSTRG.45865 | 1 | 0.67 | 1.24 | 1.16 | 0.35 | 2.12 |
|  | MSTRG.46273 | 2.15 | 2.73 | 3.05 | 3.08 | 4.48 | 4.21 |
|  | MSTRG.4655 | 1.11 | 0.74 | 0.92 | 0.85 | 1.92 | 2.73 |
|  | MSTRG.48960 | 2.79 | 2.98 | 1.84 | 2.63 | 2.74 | 2.62 |
|  | MSTRG.49066 | 1.1 | 1.61 | 0.72 | 0.82 | 1.91 | 1.44 |
|  | MSTRG.49067 | 1.01 | 0.62 | 0.43 | 0.94 | 1.47 | 0.99 |
|  | MSTRG.51853 | 0.5 | 0.96 | 2.97 | 1.17 | 2.17 | 8.03 |
|  | MSTRG.52126 | 3.56 | 8.36 | 17.8 | 8.36 | 10.01 | 14.02 |
|  | MSTRG.52498 | 3.29 | 3.41 | 2.59 | 3.97 | 3.55 | 3.86 |
|  | MSTRG.53145 | 1.31 | 0.91 | 2.87 | 0 | 0.71 | 2.68 |
|  | MSTRG.53148 | 0.55 | 0.43 | 1.72 | 1.43 | 1.63 | 3.15 |
|  | MSTRG.53161 | 2.52 | 4.32 | 8.45 | 4.52 | 4.57 | 5.17 |
|  | MSTRG.54141 | 24.77 | 36.9 | 38.06 | 26.28 | 40.85 | 30.32 |
|  | MSTRG.54142 | 0 | 0 | 0.76 | 1.55 | 0 | 0.85 |
|  | MSTRG.55877 | 13.24 | 18.1 | 22.76 | 12.28 | 21.9 | 18.12 |
|  | MSTRG.56898 | 15.69 | 8.03 | 20.26 | 15.44 | 14.24 | 16.18 |
|  | MSTRG.57409 | 0.99 | 2.51 | 2.17 | 1.35 | 1.85 | 1.17 |
|  | MSTRG.57417 | 2.29 | 6.23 | 9.13 | 4.02 | 9.5 | 20.25 |
|  | MSTRG.57419 | 0.47 | 3.45 | 2.96 | 5.72 | 6.75 | 9.86 |
|  | MSTRG.57422 | 1.89 | 1.92 | 2.45 | 2.25 | 1.46 | 2.8 |
|  | MSTRG.57652 | 0 | 0 | 0 | 0 | 1.33 | 1.39 |
|  | MSTRG.59403 | 4.79 | 4.61 | 5.51 | 9.97 | 5.19 | 3.31 |
|  | MSTRG.61020 | 5.81 | 5.9 | 7.28 | 5.48 | 4.65 | 4.91 |
|  | MSTRG.61021 | 9.65 | 10.58 | 11.22 | 10.88 | 8.32 | 7.45 |
|  | MSTRG.61233 | 0 | 0 | 0 | 0 | 0 | 0 |
|  | MSTRG.61234 | 285.55 | 272.27 | 229.29 | 273.54 | 224.93 | 186.76 |
|  | MSTRG.62702 | 3.82 | 3.02 | 3.26 | 4.2 | 4.84 | 4.24 |
|  | MSTRG.63223 | 9.1 | 9.5 | 9.88 | 10.19 | 9.74 | 10.46 |
|  | MSTRG.63297 | 3.49 | 6.27 | 4.88 | 5.47 | 5.84 | 3.01 |
|  | MSTRG.63777 | 62.47 | 68.75 | 68.73 | 73.81 | 68.58 | 64.48 |
|  | MSTRG.65882 | 3.98 | 8.63 | 13.83 | 20.95 | 15.49 | 9.99 |
|  | MSTRG.66238 | 11 | 7.49 | 7.89 | 9.36 | 10.77 | 6.82 |
|  | MSTRG.66239 | 16.07 | 6.21 | 6.41 | 9.71 | 17.96 | 4.12 |
|  | MSTRG.66334 | 0 | 4.04 | 5.35 | 1.51 | 0.72 | 5.75 |
|  | MSTRG.8549 | 15.2 | 13.27 | 15.09 | 16.87 | 15.32 | 14.02 |
| malonyl-CoA-acyl carrier protein transacylase (MCTK) | Ch_GLEAN_10006327 | 2.17 | 1.78 | 2.73 | 2.92 | 2.16 | 4.11 |
|  | Ch_GLEAN_10006911 | 16.56 | 14.55 | 13.93 | 13.99 | 17.01 | 18.79 |
|  | MSTRG.16143 | 136.17 | 200.36 | 129.47 | 164.84 | 145.32 | 115.14 |
|  | MSTRG.57652 | 0 | 0 | 0 | 0 | 1.33 | 1.39 |
| beta-ketoacyl acyl carrier protein synthase (KAS) | Ch_GLEAN_10008884 | 29.03 | 32.42 | 24.95 | 31.12 | 32.08 | 22.75 |
|  | MSTRG.26629 | 71.12 | 68.61 | 53.68 | 68.93 | 68.77 | 57.89 |
|  | MSTRG.26630 | 0.06 | 0.06 | 0.2 | 0.13 | 0.19 | 0.31 |
|  | MSTRG.61233 | 0 | 0 | 0 | 0 | 0 | 0 |
|  | MSTRG.61234 | 285.55 | 272.27 | 229.29 | 273.54 | 224.93 | 186.76 |
| acyl-acyl carrier protein thioesterase (FAFA) | Ch_GLEAN_10006981 | 11.74 | 11.05 | 11.63 | 11.42 | 12.29 | 6.84 |
|  | Ch_GLEAN_10012146 | 42.08 | 37.7 | 39.29 | 44.32 | 42.1 | 40.66 |
|  | MSTRG.66238 | 11 | 7.49 | 7.89 | 9.36 | 10.77 | 6.82 |
|  | MSTRG.66239 | 16.07 | 6.21 | 6.41 | 9.71 | 17.96 | 4.12 |
| fatty acid desaturas3 (FAD) | Ch_GLEAN_10005973 | 15.59 | 20.83 | 11.59 | 7.44 | 15.6 | 13.4 |
|  | Ch_GLEAN_10008024 | 11.86 | 28.21 | 14.16 | 11.21 | 19.62 | 14.32 |
|  | MSTRG.22936 | 0.57 | 1.24 | 1.59 | 1.68 | 3.87 | 2.61 |
|  | MSTRG.22937 | 0 | 0 | 0 | 0 | 0 | 0.84 |
|  | MSTRG.35274 | 0.16 | 0.46 | 0.56 | 1.38 | 1.54 | 1.53 |
|  | MSTRG.61836 | 0.82 | 3.25 | 1.48 | 2.11 | 4.37 | 4.88 |
| stearoyl-ACP-desaturase (SAD) | Ch_GLEAN_10011482 | 27.33 | 33.8 | 32.53 | 48.66 | 40.22 | 42.74 |
|  | MSTRG.33965 | 120.38 | 268.86 | 156.19 | 139.48 | 149.92 | 130.35 |
|  | MSTRG.33966 | 0 | 0 | 0.61 | 0 | 0.52 | 0 |
|  | MSTRG.33967 | 24.74 | 48.06 | 34.16 | 26.73 | 19.77 | 21.83 |
|  | MSTRG.47563 | 4.02 | 23.52 | 9.88 | 44.86 | 26.77 | 34.58 |
|  | MSTRG.5757 | 50.45 | 100.18 | 65.1 | 60.49 | 74.33 | 62.61 |
|  | MSTRG.59211 | 50.78 | 108 | 67.41 | 58.19 | 66.96 | 55.95 |
|  | MSTRG.59212 | 0 | 0 | 0 | 0 | 0 | 0 |
|  | MSTRG.59213 | 0 | 0 | 0 | 0 | 0 | 0 |
|  | MSTRG.64253 | 1.15 | 5.48 | 5.82 | 17.45 | 8.67 | 13.65 |

**Table S5** Primers for real time RT-PCR in this study

| Primer | Primer Sequence (5-3) | References |
| --- | --- | --- |
| PSY-F | CGATACCAGACCTTCGACG | [4] |
| PSY-R | TGCCTTATAGACCACATCCAT |  |
| PDS-F | AAGTTCAGACCCACTCAGCG | This study |
| PDS-R | AGTCCTCAACAATGGCCTCG |  |
| LYC-F | TGGAGCTGCTGCTGTCCCT | [4] |
| LYC-R | GAAGAAGAGCGTGATGCCGA |  |
| CRTR-BF | ACACCTCGCACTGGACCCT | [4] |
| CRTR-BR | GTATAGCGTGATGCCCAGCC |  |
| BKT-F | CAATCTTGTCAGCATTCCGC | [4] |
| BKT-R | CAGGAAGCTCATCACATCAGAT |  |
| IPI-1-F | GCGAGCACGAAATGGACTAC | [4] |
| IPI-1-R | GCTGCATCATCTGCCGCA |  |
| IPI-2-F | AGTACCTGGCGCAAAAGCTG | [4] |
| IPI-2-R | GTTGGCCCGGATGAATAAGA |  |
| CRTO-F | ACGTACATGCCCCACAAG | [4] |
| CRTO-R | CAGGTCGAAGTGGTAGCAGGT |  |
| BC-F | CAAGAAGGTGATGATCGCCA | [33] |
| BC-R | GACGTGCAGCGAGTTCTTGTC |  |
| ACP-F | CAGCTCGGCACTGACCTTG | [33] |
| ACP-R | CAAGGGTCAGCTCGAACTTCTC |  |
| MCTK-F | GGTGAGGACAAGGCGGTG | [33] |
| MCTK-R | TCATCCTGGCCTTGAAGCTC |  |
| KAS-F | CACCCCACTCTGAACCAGGA | [33] |
| KAS-R | GACCTCCAAACCCGAAGGAG |  |
| FATA-F | AGACTCGTTCAGCGAGGAGC | [33] |
| FAFA-R | CATGCCCACAGCATGGTTC |  |
| SAD-F | CCGAGCCCAAGCTTCTAGTG | [33] |
| SAD-R | TTTGCCTCCATGTAATCCCC |  |
| FAD-F | GTAGGTCACCACGTCCAGCC | [33] |
| FAD-R | CTTGATAGGCATGCTGGGTGT |  |
| Actin-F | ACCTCAGCGTTCAGCCTTGT | [33] |
| Actin-R | TGGTCCACGACACCATCAAC |  |
